# Inhibition of nucleolar stress response by Sirt1: A potential mechanism of acetylation‐independent regulation of p53 accumulation

**DOI:** 10.1111/acel.12900

**Published:** 2019-01-08

**Authors:** Xiaolei Bi, Qing Ye, Daoyuan Li, Qisheng Peng, Zhe Wang, Xiao Wu, Yun Zhang, Qunye Zhang, Fan Jiang

**Affiliations:** ^1^ School of Basic Medicine Shandong University Jinan Shandong Province China; ^2^ Key Laboratory of Cardiovascular Remodeling and Function Research Chinese Ministry of Education, Chinese National Health Commission and Chinese Academy of Medical Sciences Jinan China; ^3^ The State and Shandong Province Joint Key Laboratory of Translational Cardiovascular Medicine, Department of Cardiology Qilu Hospital of Shandong University Jinan China; ^4^ National Glycoengineering Research Center Shandong University Jinan China; ^5^ Key Laboratory of Zoonosis Research Jilin University Changchun Jilin Province China; ^6^ Division of Endocrinology and Metabolism Shandong Provincial Hospital affiliated to Shandong University Jinan China; ^7^ Present address: Department of Cardiology Qingdao Municipal Hospital Qingdao Shandong Province China

**Keywords:** acetylation, nucleolar stress response, nucleophosmin, p53, SILAC, Sirt1

## Abstract

The mammalian Sirt1 deacetylase is generally thought to be a nuclear protein, but some pilot studies have suggested that Sirt1 may also be involved in orchestrating nucleolar functions. Here, we show that nucleolar stress response is a ubiquitous cellular reaction that can be induced by different types of stress conditions, and Sirt1 is an endogenous suppressor of nucleolar stress response. Using stable isotope labeling by amino acids in cell culture approach, we have identified a physical interaction of between Sirt1 and the nucleolar protein nucleophosmin, and this protein–protein interaction appears to be necessary for Sirt1 inhibition on nucleolar stress, whereas the deacetylase activity of Sirt1 is not strictly required. Based on the reported prerequisite role of nucleolar stress response in stress‐induced p53 protein accumulation, we have also provided evidence suggesting that Sirt1‐mediated inhibition on nucleolar stress response may represent a novel mechanism by which Sirt1 can modulate intracellular p53 accumulation independent of lysine deacetylation. This process may represent an alternative mechanism by which Sirt1 regulates functions of the p53 pathway.

## 1 INTRODUCTION

The mammalian NAD^+^‐dependent deacetylase Sirt1 is a crucial regulator of cell stress response and cell senescence. Sirt1 is activated under metabolic stress conditions, and activation of Sirt1 orchestrates functional changes of multiple metabolic pathways, which are important for cellular adaptation to nutrient deprivation (Canto & Auwerx, 2012; Chalkiadaki & Guarente, 2012). Sirt1 is also activated by reactive oxygen species (ROS; Chung et al., 2010), which endows cellular tolerance against oxidative stress‐induced injuries (Chong, Shang, Wang, & Maiese, 2012). Moreover, Sirt1 has profound effects in preventing cell aging. Inhibition of Sirt1 promotes senescence, whereas increased Sirt1 function retards the development of cell senescence (Huang et al., 2008; Langley et al., 2002; Ota et al., 2007; Yamashita et al., 2012; Zu et al., 2010). Consistent with this effect, Sirt1 exhibits multiple beneficial actions in various aging‐related pathologies in vivo (Chalkiadaki & Guarente, 2012; Hall, Dominy, Lee, & Puigserver, 2013; Herranz & Serrano, 2010).

A potential mechanism by which Sirt1 inhibits cell senescence is the repression of functions of the tumor suppressor p53 (Langley et al., 2002; Ota et al., 2007; Yi & Luo, 2010), which has a pivotal role in mediating cell senescence (Yi & Luo, 2010). Sirt1 catalyzes deacetylation of K382 at the C‐terminus of p53 (Vaziri et al., 2001), causing decreased DNA binding ability and transcriptional activity (Luo et al., 2004; Tang, Zhao, Chen, Zhao, & Gu, 2008; Vaziri et al., 2001). Sirt1 also promotes p53 protein degradation; when Sirt1 expression is knocked down, cells exhibit increased protein levels of p53 (Ford, Jiang, & Milner, 2005). However, how Sirt1 regulates p53 stabilization is not totally understood. Although there is evidence showing that acetylation of the C‐terminus of p53 is sufficient to promote protein stabilization (Li, Luo, Brooks, & Gu, 2002), different studies have suggested that deacetylation of K382 by Sirt1 may not have a major impact on the rate of p53 degradation (Nakamura, Roth, & Mukhopadhyay, 2000; Rodriguez, Desterro, Lain, Lane, & Hay, 2000). Moreover, both in vitro and in vivo experiments have demonstrated that simultaneous mutation of all of the C‐terminal lysine residues fails to abrogate stress‐induced p53 stabilization (Feng, Lin, Uranishi, Gu, & Xu, 2005; Krummel, Lee, Toledo, & Wahl, 2005), indicating uncoupling of p53 acetylation and stabilization (Cheng et al., 2003). All these data strongly suggest that a deacetylation‐independent mechanism may exist, which is responsible for Sirt1‐induced p53 degradation.

Mounting evidence has suggested that the nucleolus, in addition to its primary function in ribosome biogenesis, is a signaling hub involved in mediating cellular stress responses (which is termed nucleolar stress; Boulon, Westman, Hutten, Boisvert, & Lamond, 2010; James, Wang, Raje, Rosby, & DiMario, 2014; Tsai & Pederson, 2014; Vlatkovic, Boyd, & Rubbi, 2014). Nucleolar stress response (NSR) is characterized by disorganization of the normal nucleolar structure and translocation of some nucleolar proteins to the nucleoplasm, for example, nucleolin, fibrillarin, nucleophosmin/B23 (NPM), and nucleostemin (Avitabile et al., 2011; Boulon et al., 2010; Chan, 1992; Chen & Jiang, 2004; Daniely, Dimitrova, & Borowiec, 2002). Functionally, induction of nucleolar stress results in p53 stabilization, likely by disruption of the p53 binding to the E3 ubiquitin ligase MDM2, leading to decreased p53 ubiquitination and degradation (James et al., 2014; Tsai & Pederson, 2014; Vlatkovic et al., 2014). Occurrence of the NSR may be implicated in the pathogenesis of neurodegenerative and cardiovascular diseases (Avitabile et al., 2011; Rieker et al., 2011; Tsoi, Lau, Tsang, Lau, & Chan, 2012). Interestingly, it has been observed that irradiation‐induced DNA damage per se is not able to trigger p53 accumulation, unless the nucleolus is disrupted; moreover, forced nucleoli disruption in the absence of genotoxic stress also causes p53 stabilization, indicating that NSR is both sufficient and necessary for p53 stabilization (Rubbi & Milner, 2003). Further studies also confirmed an obligatory role of nucleoli in controlling p53 accumulation (Boyd, Vlatkovic, & Rubbi, 2011).

The subcellular localization of Sirt1 may have significant impacts on the roles of Sirt1 in cell biology (Song & Surh, 2012). Sirt1 is generally recognized as a nuclear protein, although it is also present in the cytosol (Nogueiras et al., 2012). Intriguingly, some studies have suggested that Sirt1 may also have a role in the nucleoli (Murayama et al., 2008; Song et al., 2013; Straight et al., 1999; Yang et al., 2013). For instance, Murayama and colleagues have demonstrated that Sirt1 is a partner of a nucleolar protein complex termed eNoSC (energy‐dependent nucleolar silencing complex), which senses intracellular energy status and controls rRNA transcription (Murayama et al., 2008). Under energy stress, Sirt1 in the eNoSC coordinates with the methyltransferase SUV39H1 to repress rRNA transcription and facilitates restoration of the energy homeostasis (Murayama et al., 2008). Similarly, Yang et al. showed that nutrient deprivation stimulated Sirt1 binding to another eNoSC component nucleomethylin, which also contributed to repression of rRNA transcription (Yang et al., 2013). These data raise a question of whether Sirt1 is involved in modulating cellular NSR. In the present study, using a stable isotope labeling by amino acids in cell culture (SILAC)‐based proteomics approach (Gruhler & Kratchmarova, 2008), we identified a physical interaction between Sirt1 and NPM. Moreover, we show that Sirt1 is an endogenous inhibitor of NSR. Our results may provide a plausible explanation to the observed Sirt1‐mediated, deacetylation‐independent regulation of p53 stabilization.

## 2 RESULTS

### 2.1 NSR is a ubiquitous cellular reaction to multiple stress stimuli

**Figure 1:**
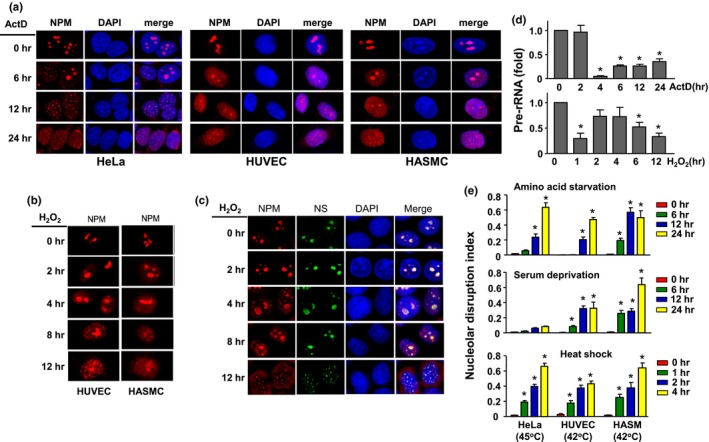
Induction of nucleolar stress response (NSR) in both immortalized and primary cells. (a) NSR induced by actinomycin D (ActD) of 5 nM in HeLa, human umbilical vein endothelial (HUVEC) and human aortic smooth muscle (HASMC) cells, as detected by nucleophosmin (NPM) immunofluorescence. Nuclei were stained with DAPI. (b) NSR induced by H_2_O_2_ in HUVECs (at 300 µM) and HASMC (at 600 µM). (c) H_2_O_2_ (600 µM)‐induced NSR in HeLa as revealed by dislocation of NPM and nucleostemin (NS). (d) Effects of ActD and H_2_O_2_ on the expression levels of pre‐rRNA in HeLa cells. (e) Induction of NSR by various stimuli as indicated, assessed by nucleolar disruption index which was defined as the nucleoplasm to nucleoli ratio of the average fluorescence intensity. Data are expressed as mean ± *SEM*. *p < 0.05 versus control, one‐way ANOVA, *n* = 3–5

In this study, we first characterized the presence of NSR in both transformed and primary cells using divergent stimuli. Actinomycin D (ActD) of low concentrations (e.g., <10 nM) was the prototype inducer of nucleolar stress via specific inhibition of the RNA polymerase I and rDNA transcription (Deisenroth & Zhang, 2010). Using NPM dislocation as a marker, we confirmed that ActD at 5 nM triggered NSR not only in HeLa cells, but also in primary HUVEC and HASMC cells (Figure 1a). In HeLa cells pretreated with ActD for 24 hr, removal of ActD did not re‐establish the normal NPM morphology up to 24 hr, indicating that prolonged NSR was irreversible (data not shown). Although ActD is an established NSR inducer, it has little relevance to most diseases. We therefore tested the effects of oxidative stress using exogenous H_2_O_2_. We showed that oxidative stress consistently induced NSR in all of the cells (Figure 1b,c). We further confirmed the NSR in response to H_2_O_2_ by detecting nucleostemin localization. Nucleostemin showed a high degree of co‐localization with NPM in the nucleoli. Upon H_2_O_2_ treatment, nucleostemin exhibited similar dislocation from nucleoli to the nucleoplasm, which occurred later than that of NPM (Figure 1c). Moreover, we demonstrated that both ActD and H_2_O_2_ similarly reduced the level of pre‐rRNA, indicating compromised rDNA transcriptions (Figure 1d). We next asked whether NSR might represent a ubiquitous stress response to various stimuli; hence, we treated the cells with amino acid starvation, serum deprivation, and heat shock. Amino acid starvation induced typical NSRs in all of the cells. Serum deprivation induced NSRs only in HUVECs and HASMCs. Heat shock at 42°C induced NSRs in HUVECs and HASMCs but not in HeLa cells, while heat shock at 45°C also induced NSR in HeLa cells (Figure 1e). It was noted that there were some variations in the time course of the induction of NSR by each stimuli in different cells. Generally, transformed cells were more resistant to the induction of NSR than primary cells. In all of the above experiments, the specific stress conditions applied were optimized by ensuring that they did not cause obvious cell death at least within 48 hr.

### 2.2 Sirt1 is an endogenous inhibitor of NSR

**Figure 2:**
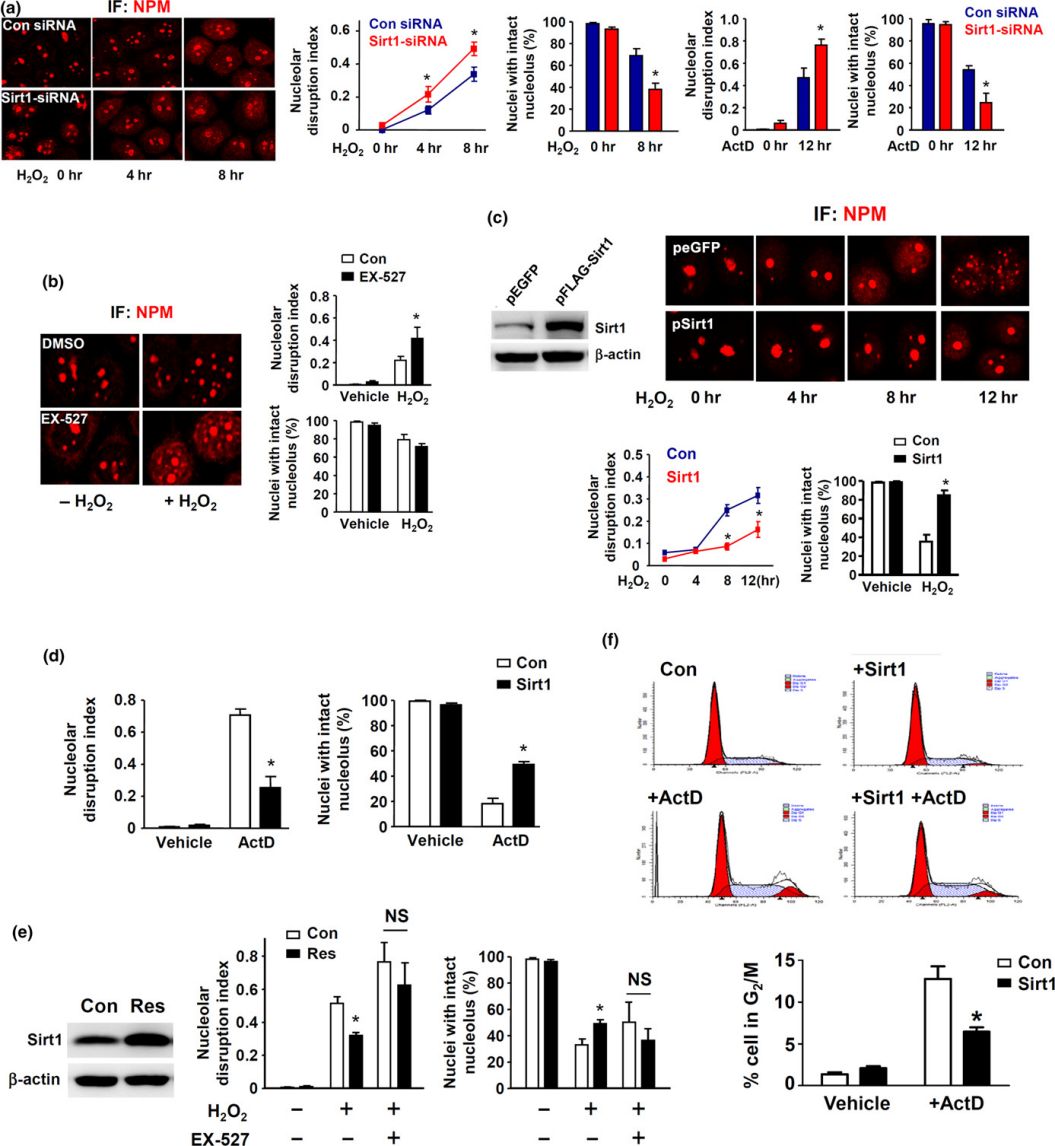
Sirt1 has inhibitory effects on nucleolar stress response (NSR). (a) Effects of Sirt1 gene silencing on NSR induced by ActD (5 nM) and H_2_O_2_ (600 µM), assessed by nucleophosmin/B23 immunofluorescence. (b) Effects of the Sirt1 inhibitor EX‐527 (10 μM) on H_2_O_2_‐induced NSR. (c and d) Effects of Sirt1 overexpression (pFlag‐SIRT1) on H_2_O_2_‐ and ActD‐induced NSR. (e) Treatment with the Sirt1 activator resveratrol (10 µM) significantly increased the protein level of Sirt1 and suppressed H_2_O_2_‐induced NSR, while the NSR inhibiting effect of resveratrol was blunted by EX‐527. (f) Flow cytometry data showing that Sirt1 overexpression could inhibit ActD‐induced G2/M cell cycle blockade. All experiments were performed in HeLa cells. *p < 0.05 versus control (Con), one‐way ANOVA, *n* = 3–5. NS, no significance; IF, immunofluorescence

In the following experiments, we used ActD and H_2_O_2_ as the stimuli to investigate whether Sirt1 had any role in regulating NSR. We first knocked down Sirt1 expression with siRNA in HeLa cells as used in our previous studies (Liu et al., 2015, 2013). We showed that Sirt1 siRNA significantly accelerated the occurrence of nucleolar stress induced by both ActD and H_2_O_2_ (Figure 2a). Next, we pretreated cells with the specific Sirt1 inhibitor EX‐527. EX‐527 alone did not trigger NSR, but increased NPM dislocation induced by H_2_O_2 _(Figure 2b). However, unlike Sirt1 silencing, EX‐527 had no significant effects on the proportion of cells with disintegrated nucleoli (Figure 2b). To further establish the role of Sirt1, we overexpressed human wild‐type Sirt1 in HeLa cells (Figure 2c) followed by the treatment with H_2_O_2_. Sirt1 overexpression significantly attenuated the development of NSR induced by H_2_O_2_ (Figure 2c). We confirmed that Sirt1 overexpression also significantly attenuated NSR induced by ActD (Figure 2d). Finally, we treated the cells with the Sirt1 activator resveratrol. Consistent with our previous findings in endothelial cells (Liu et al., 2015), we showed that resveratrol significantly augmented the expression level of Sirt1 in HeLa cells (Figure 2e). Treatment with resveratrol attenuated NSR induced by H_2_O_2_, while this effect of resveratrol was blunted by EX‐527 (Figure 2e). We and others have shown that NSR induction by inhibiting RNA polymerase I triggers cell cycle arrest (Negi & Brown, 2015; Ye et al., 2017). To test whether Sirt1 could also inhibit NSR‐associated cellular functional changes, we performed flow cytometry analysis; we showed that ActD treatment caused G2/M blockade, which was attenuated by Sirt1 overexpression (Figure 2f).

### 2.3 Sirt1‐dependent inhibition against NSR revealed by live cell imaging

To monitor the dynamic changes of nucleoli integrity under stress conditions and the effects of Sirt1, we co‐transfected the cells with peGFP/FLAG‐NPM plus either pFLAG‐Sirt1 or a control vector, and treated the cells with ActD. In resting cells, the eGFP signal showed clear nucleolar localization (Supporting information Figure S1). ActD‐treated cells exhibited diffusion of the eGFP signal from nucleoli to the nucleoplasm, and this process was inhibited by Sirt1 overexpression (Supporting information Figure S1). Interestingly, we observed that a proportion of the cells entered cell cycle even in the presence of ActD. In normal dividing cells, the nucleoli disassembled during the mitotic phase and reassembled in the early G1 phase as described (Hernandez‐Verdun, Roussel, & Gebrane‐Younes, 2002). In ActD‐treated cells, however, the disassembled nucleoli did not reappear in the daughter cells by the end of the experiment. In Sirt1‐overexpressing cells, reformation of nucleoli at the end of cell division remained to be observable in the presence of ActD.

### 2.4 Sirt1 physically interacts with NPM

**Figure 3:**
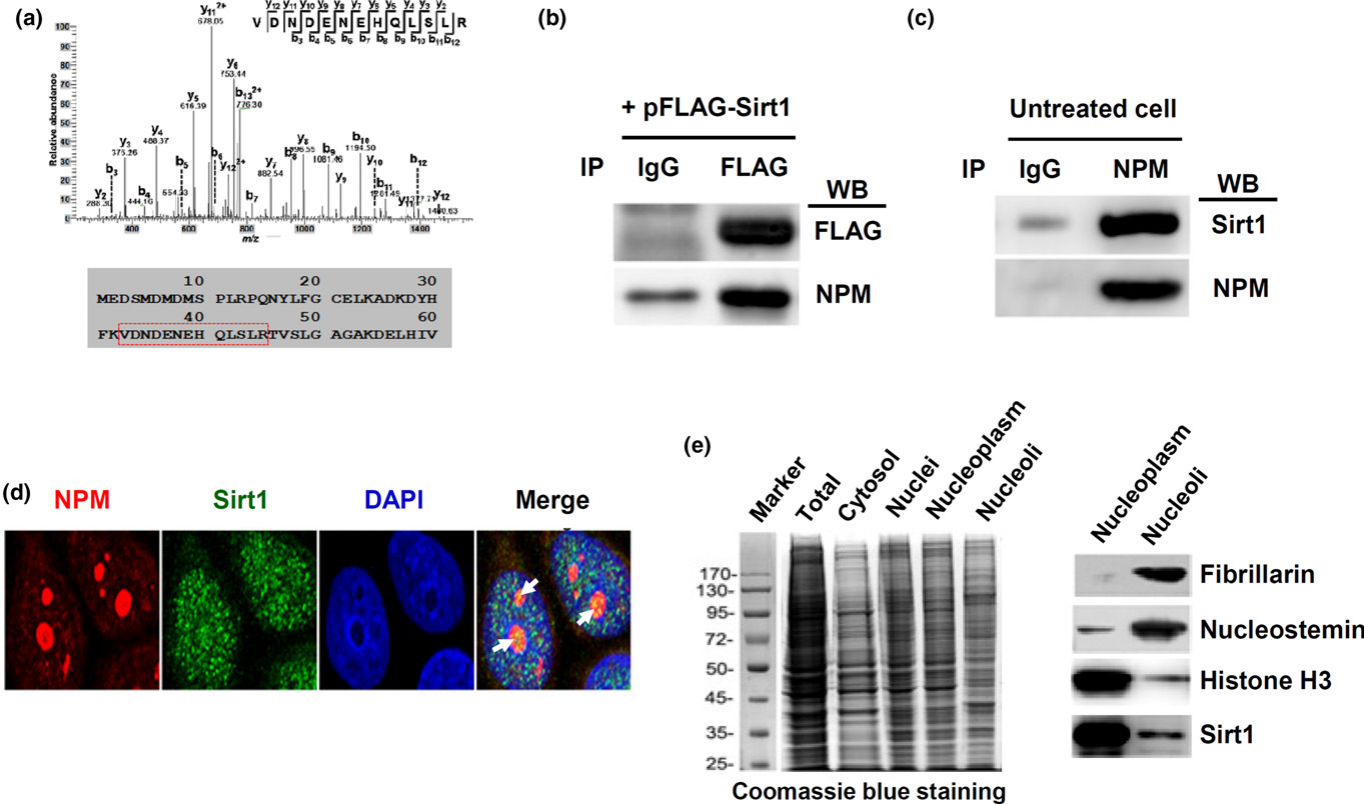
Physical interaction between Sirt1 with nucleophosmin (NPM). (a) Representative mass spectrogram of a peptide segment of NPM (sequence indicated by the red box below) co‐immunoprecipitated with Sirt1. (b) Immunoprecipitation (IP) of FLAG‐Sirt1 fusion protein in transfected cells and Western blot (WB) detection of bound NPM. Normal IgG was used in IP as a negative control. (c) Immunoprecipitation of endogenous NPM in untreated cells and WB detection of bound Sirt1. (d) Fluorescent confocal microscopic images showing partial co‐localization of NPM with Sirt1 (yellow color as indicated by the arrows) in the nucleolar compartment. DAPI was used to stain the nucleus. (e) Western blot detection of Sirt1 protein in purified nucleoli (right panel). Fibrillarin and nucleostemin were nucleolar markers. Histone H3 was a nuclear marker (which was also present in nucleoli to a lesser extent). The left panel showed a Coomassie blue‐stained PAGE gel showing the electrophoresis patterns of various subcellular fractions as indicated. All experiments were performed using HeLa cells

To identify the potential mechanisms of how Sirt1 regulated nucleolar stress, we transfected HeLa cells with pFLAG‐Sirt1 and performed SILAC‐based proteomic analysis for Sirt1 binding partners. Among the more than 200 proteins detected, there are a number of known Sirt1 substrates, including tubulin, histones H3 and H4, STAT3, and 14‐3‐3 protein, indicating that this approach was successful. We also detected multiple ribosomal proteins and proteins involved in RNA processing, including various hnRNPs, snRNPs, and splicing factors (Supporting information Table S1). One of the Sirt1 binding partners was the nucleolar protein NPM (Figure 3a), which had a critical role in maintaining nucleoli integrity (Amin, Matsunaga, Uchiyama, & Fukui, 2008). Therefore, we further investigated the interactions between Sirt1 and NPM. Co‐immunoprecipitation experiments in pFLAG‐Sirt1 transfected cells showed that NPM bound to the ectopically expressed Sirt1 (Figure 3b). To further confirm the binding of NPM with endogenous Sirt1, we immunoprecipitated NPM in untreated cells, and detected the bound form of Sirt1 with Western blot (Figure 3c). We also identified partial co‐localization of NPM and Sirt1 in the nucleolar compartment using fluorescent confocal microscopy (Figure 3d). Moreover, we isolated cell nuclei and purified the nucleoli (Figure 3e). We demonstrated that although the majority of Sirt1 was located in the nucleoplasm, a small portion of Sirt1 was also present in the nucleolar fraction (Figure 3e).

### 2.5 The effect of Sirt1 is partially independent of its deacetylase activity

We showed that the Sirt1 inhibitor EX‐527 facilitated stress‐induced NSR. To clarify whether the action of Sirt1 was totally dependent on its deacetylase activity, we compared the effects of wild‐type Sirt1 and Sirt1‐H363Y. Interestingly, we found that Sirt1‐H363Y also exhibited an inhibitory effect on ActD‐induced NSR, although this effect appeared to be smaller than that of the wild‐type Sirt1 (Supporting information Figure S2a). To further confirm this observation, we repeated the experiments in cells pretreated with EX‐527. We demonstrated that both wild‐type Sirt1 and Sirt1‐H363Y produced inhibitory effects on NSR in the presence EX‐527, and in contrast to their effects in EX‐527 untreated cells, there was no significant difference between the two treatment groups (Supporting information Figure S2b).

### 2.6 The effect of Sirt1 on NSR is not associated with deacetylation of the C‐terminal domain of NPM

**Figure 4:**
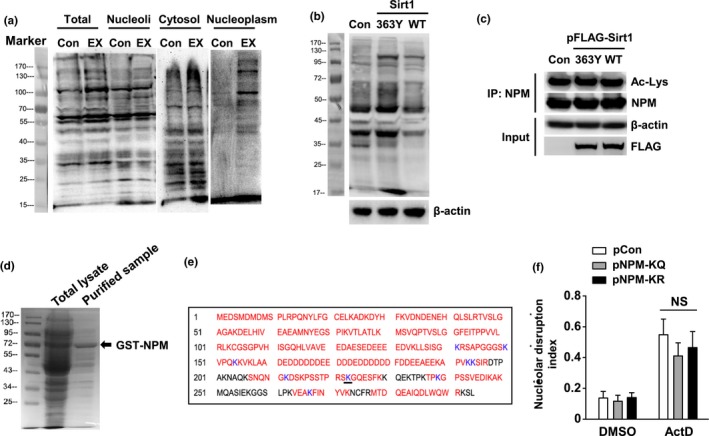
The inhibitory effect of Sirt1 on nucleolar stress response (NSR) was not associated with deacetylation of nucleophosmin (NPM). (a) Western blot showing changes in protein acetylation profiles in various subcellular fractions from untreated and EX‐527 (10 μM)‐treated cells detected by the anti‐acetyl lysine antibody (clone 15G10). (b) Expression of wild‐type Sirt1 (WT), but not Sirt1‐H363Y, reduced the overall level of protein acetylation assayed with whole cell homogenates. The moderate enhancing effect of Sirt1‐H363Y might be a dominant‐negative effect. (c) Immunoprecipitation (IP) and Western blot results showing that the level of NPM acetylation detected using the anti‐acetyl lysine (Ac‐Lys) antibody was not affected by expression of either wild‐type or H363Y mutant of Sirt1. (d) Coomassie blue‐stained PAGE gel showing the band of purified GST‐NPM fusion protein expressed in *E. coli*, which was used for in vitro acetylation–deacetylation and mass spectrometry assays. (e) Diagram showing the lysine residues in human NPM that could be acetylated in vitro (blue letters). Mass spectrometry confirmed that only K223 (underlined) was deacetylated by incubation with recombinant Sirt1. Black letters indicated segments which were not covered by the mass spectrometry. (f) Overexpression of K223Q or K223R NPM mutant did not exhibit significant effects on ActD‐induced NSR in ectopic eGFP‐NPM expressing HeLa cells. All experiments were performed using HeLa cells. Data are mean ± *SEM*, *n* = 3. NS, no significance

Previous studies have shown that the C‐terminal domain of NPM is essential for its DNA binding activity (Hingorani, Szebeni, & Olson, 2000). Mutations in this region of NPM, which underlie a subgroup of acute myeloid leukemia, result in aberrant dislocation of NPM from the nucleoli to nucleoplasm and cytosol (Falini, Nicoletti, Martelli, & Mecucci, 2007). Moreover, there is evidence showing that multiple C‐terminal lysine residues in NPM undergo CBP/p300‐dependent acetylation (Shandilya et al., 2009; Swaminathan, Kishore, Febitha, & Kundu, 2005). Hence, we tested whether the NSR inhibiting effect of Sirt1 was associated with alterations of NPM deacetylation. We first performed immunoprecipitation and Western blot experiments using a monoclonal anti‐acetyl lysine antibody. The antibody was validated by probing lysates of cells with and without EX‐527 treatment (Figure 4a), showing that EX‐527 increased the overall levels of protein acetylation in various subcellular fractions. Next, we demonstrated that expression of wild‐type Sirt1, but not Sirt1‐H363Y, reduced the level of protein acetylation (Figure 4b). However, we found that overexpression of either wild‐type Sirt1 or Sirt1‐H363Y had no effect on the level of NPM acetylation (Figure 4c). To confirm this negative finding, we prepared recombinant GST‐NPM fusion protein and performed in vitro acetylation assays (Figure 4d,e). Using Orbitrap‐based mass spectrometry analysis, we confirmed that all of the detected lysine residues in native NPM were nonacetylated. We identified that K193, K194, K212, K223, K239 and K267 could be acetylated by CBP/p300 in vitro. Subsequent treatment with recombinant human Sirt1 protein only induced deacetylation of K223. These data indicate that deacetylation of NPM is unlikely to be essential for the NSR inhibiting action of Sirt1. To further exclude the possibility that NPM K223 acetylation was involved in modulating NSR, we overexpressed K223Q or K223R NPM mutant and treated the cells with ActD. We showed that NPM‐K223Q or NPM‐K223R did not exhibit significant effects on ActD‐induced NSR (Figure 4f).

### 2.7 Role of Sirt1‐NPM binding in NSR inhibition

**Figure 5:**
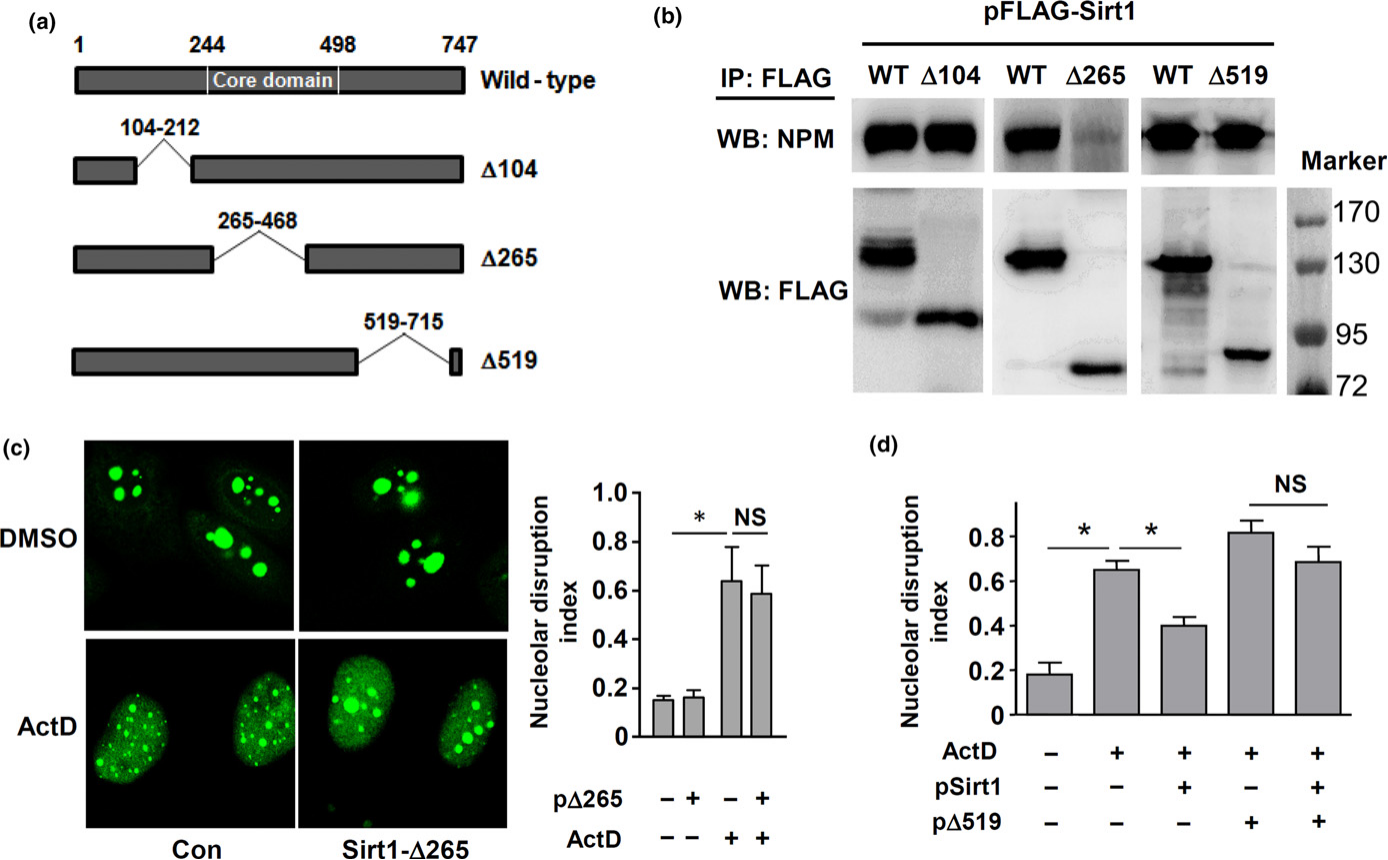
Sirt1 binding to nucleophosmin (NPM) was required for its inhibitory effect on Nucleolar stress response (NSR). (a) Diagram showing constructs of different Sirt1 deletion mutants. (b) Binding between NPM and deletion mutants of Sirt1. Cells expressing FLAG‐tagged wild‐type or mutant Sirt1 were immunoprecipitated (IP) with anti‐FLAG antibody. Bound NPM was detected by Western blot (WB). (c) Overexpression of Sirt1‐∆265 exhibited no inhibitory effect on ActD‐induced NSR in eGFP‐NPM expressing cells. (d) Co‐expression of Sirt1‐∆519 blunted the inhibitory effect of wild‐type Sirt1 on ActD‐induced NSR in eGFP‐NPM expressing cells. All experiments were performed using HeLa cells. *p < 0.05, one‐way ANOVA, *n* = 3–4. NS, no significance

Given that deacetylation of NPM did not have a major role in modulating NSR, we then examined whether the physical interaction between Sirt1 and NPM was involved. We created serial deletion mutants of Sirt1 (Figure 5a). We showed that deletion of a portion of ~200 amino acids located in the core catalytic domain of Sirt1 (∆265) abolished the binding between Sirt1 and NPM (Figure 5b). In contrast, deletion of a portion close to the N‐ or C‐terminus of Sirt1 (∆104 and ∆519) did not affect the binding. Overexpression of Sirt1‐∆265 exhibited no inhibitory effect on ActD‐induced NSR (Figure 5c). However, we observed that neither ∆104 (data not shown) nor ∆519 showed any inhibitory effect on NSR (Figure 5d), indicating that Sirt1‐NPM binding was a necessary but not a sufficient condition for NSR inhibition. Based on this finding, we next utilized ∆519 as a dominant‐negative inhibitor to further confirm the requirement of Sirt1‐NPM binding for NSR inhibition. We showed that in cells overexpressing ∆519, transfection of wild‐type Sirt1 failed to inhibit NSR (Figure 5d), indicating that blocking normal Sirt1‐NPM binding with ∆519 abolished the NSR inhibiting action of Sirt1.

### 2.8 Sirt1 may induce p53 accumulation in a deacetylation‐independent manner

**Figure 6:**
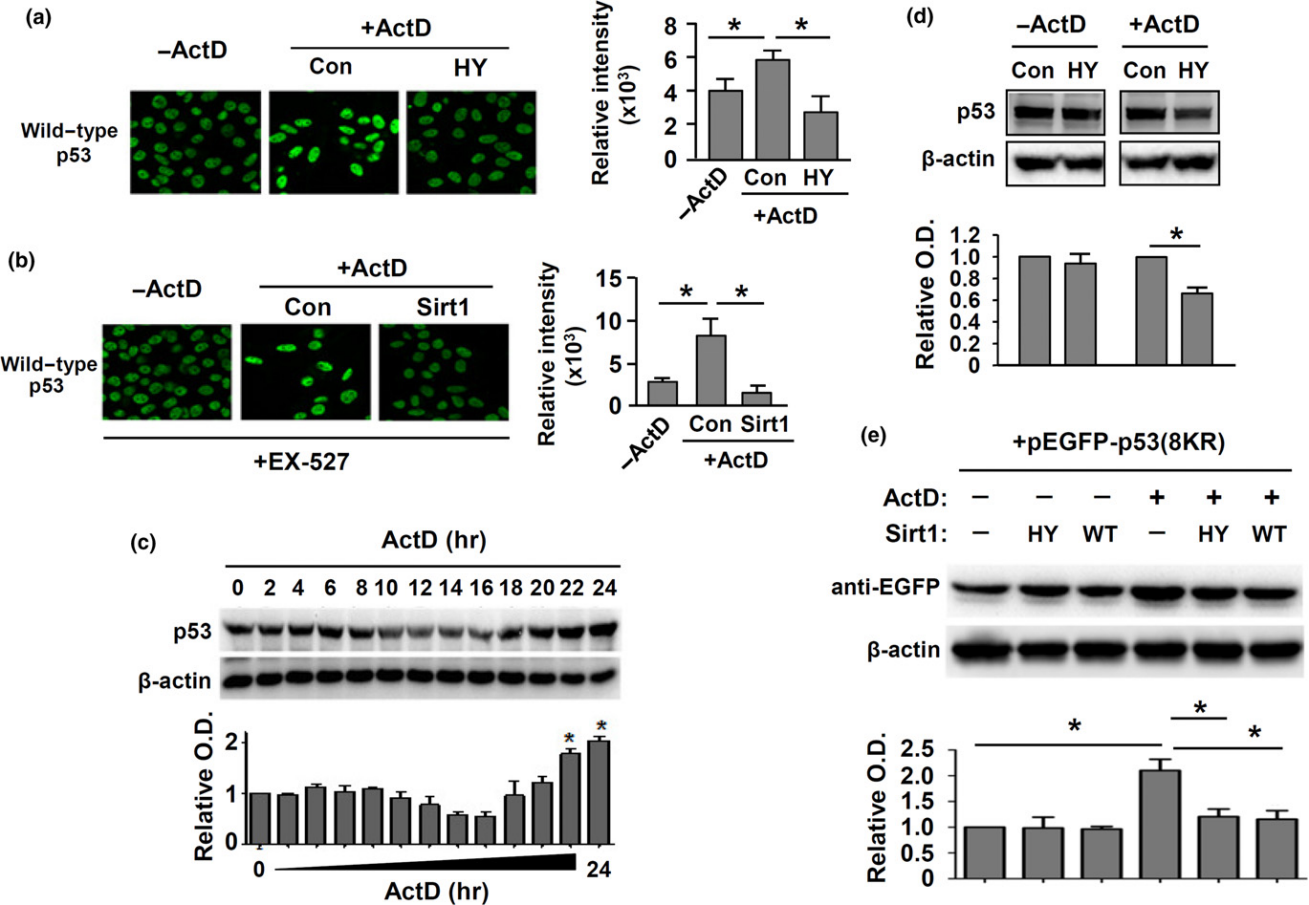
Deacetylation‐independent modulation of p53 accumulation by Sirt1. (a) Immunofluorescence of p53 and the quantitative data showing the effect of H363Y mutant (HY) of Sirt1 on ActD‐induced p53 accumulation (localized primarily in the nuclei). (b) Immunofluorescence of p53 showing the effect of wild‐type Sirt1 on ActD‐induced p53 accumulation in EX‐527 pretreated cells. (c) Western blot and quantitative densitometry data showing the time course of ActD‐induced p53 accumulation. (d) Western blots showing the effect of Sirt1‐H363Y on p53 protein level in resting and ActD‐treated cells. (e) Effects of wild‐type Sirt1 (WT) and Sirt1‐H363Y (HY) on ActD‐induced accumulation of ectopically expressed eGFP‐p53‐8KR (Western blot was performed using an anti‐eGFP antibody). All experiments were performed using HeLa cells. *p < 0.05, one‐way ANOVA, *n* = 3–5

It has been demonstrated that acetylation‐independent p53 stabilization in response to stress stimuli can occur both in vitro and in vivo (Feng et al., 2005; Krummel et al., 2005). Moreover, NSR has been shown to be a sufficient and necessary condition for p53 stabilization (Rubbi & Milner, 2003). Based on these observations, our data strongly suggest that Sirt1‐mediated inhibition of NSR may be an additional mechanism of (de)acetylation‐independent p53 regulation. Nonetheless, direct experimental evidence of Sirt1‐induced, deacetylation‐independent effect on p53 accumulation is still lacking. To test this, we first showed that overexpression of Sirt1‐H363Y produced inhibitory effects on ActD‐induced p53 accumulation (primarily in the nuclei; Figure 6a). Next, we pretreated cells with EX‐527; we found that Sirt1 overexpression could reduce ActD‐induced p53 accumulation in the presence of EX‐527 (Figure 6b). The effect of EX‐527 alone on p53 was insignificant. In both experiments, the effects of either WT or H363Y Sirt1 in ActD‐untreated cells were minor. To confirm these results, we performed Western blot tests on p53. Time course analysis showed that ActD‐induced p53 accumulation occurred later (at 22–24 hr) than the appearance of NSR (before 12 hr), as shown in Figure 6c. Overexpression of Sirt1‐H363Y significantly reduced the total p53 level in ActD‐treated cells (but not in resting cells; Figure 6d). Moreover, we expressed eGFP‐tagged p53‐8KR mutant, and demonstrated that both of wild‐type and H363Y Sirt1 reduced the level of p53‐8KR in ActD‐treated cells (Figure 6e). We also confirmed that overexpression of Sirt1‐H363Y produced 45.5% ± 6.9% reduction of p53 fluorescence intensity in HeLa cells stimulated with H_2_O_2_ (600 µM; *p* < 0.05, *n* = 4).

## 3 DISCUSSION

Emerging evidence supports that NSR is a ubiquitous cellular stress response, rather than a nonspecific cytotoxic phenomenon (Yang, Yang, & Yi, 2018). Here, we have confirmed that NSR can be induced by different types of stress stimuli in both transformed and primary cells. Using both gain‐ and loss‐of‐function experiments, here we have shown that Sirt1 is an endogenous suppressor of NSR. In the absence of a NSR inducer, however, loss of Sirt1 is not sufficient to initiate NSR, suggesting that the NSR modulating effect of Sirt1 is only important under stress conditions. The biological significance of cellular NSR is still poorly understood. NSR has been found to be associated with pathogenesis of certain cardiovascular and neurodegenerative diseases (Hariharan & Sussman, 2014; Parlato & Liss, 2014). On the other hand, it has also been recognized that NSR may represent an important signaling module leading to accumulation of p53 and cell cycle arrest, and this function may have anti‐tumorigenic effects (James et al., 2014). Therefore, we suggest that NSR is functionally a double‐edged sword; whether the inhibitory effect of Sirt1 on NSR represents a protective or deleterious process needs to be considered in a context‐dependent manner.

The precise mechanism of the inhibitory effect of Sirt1 on NSR is not clear. We have shown that the NSR inhibiting effect of Sirt1 is partially independent of its deacetylase activity, while the physical interaction between Sirt1 and NPM appears to be required for this action. Consistent with these observations, we demonstrate that Sirt1 does not exhibit major impacts on the acetylation status of NPM, although deacetylation of a single lysine residue (K223) is observed. Further mutation experiments also suggest that the status of K223 acetylation is not crucial for NSR induction. Our results indicate that the N‐terminal part of the core domain of Sirt1 is required for NPM binding, and this finding is supported by previous studies showing that this region is also responsible for interactions with other Sirt1 partners such as DBC1 (Deleted in Breast Cancer 1) and PGC‐1α (Peroxisome Proliferator‐Activated Receptor γCoactivator‐1α; Kim, Chen, & Lou, 2008; Nemoto, Fergusson, & Finkel, 2005). Moreover, our results are consistent with those from a recent study showing that NPM can also interact with Sirt6 and Sirt7 (Lee et al., 2014), which have much shorter N‐ and C‐terminal regions flanking the conserved catalytic core domain than Sirt1 (Flick & Luscher, 2012). Indeed, several studies suggest that in certain circumstances, Sirt1 may act as an adaptor molecule that facilitates the association of other members of a functional protein complex (Liang et al., 2008; Nemoto et al., 2005). Given the crucial roles of NPM in maintaining nucleolar homeostasis (Amin et al., 2008; Mitrea et al., 2016), we argue that Sirt1 may inhibit NSR by binding to NPM and preventing its dislocation, while concomitant deacetylation of other NPM‐binding partners may facilitate the NPM functions. Upon binding to NPM, an intact deacetylase activity may further enhance the effect of Sirt1 by deacetylating other binding partners. Interestingly, a recent study has proposed a similar signaling model, in which Sirt1 regulates distribution and enzymatic activity of APE1 (apurinic/apyrimidinic endonuclease 1), a partner of NPM within the nucleolus (Lirussi et al., 2012). On the other hand, many studies have pointed to a critical role of Sirt7 in maintaining the normal structure and/or function of the nucleolus (Chenet al., 2016, 2013; Paredes et al., 2018). Interestingly, Sirt7 deficiency leads to nucleolar fragmentation and rDNA heterochromatin instability, and the nucleolar protective functions of Sirt7 in turn require a synergy with Sirt1 (Ianni, Hoelper, Krueger, Braun, & Bober, 2017). Although we did not detect Sirt7 co‐immunoprecipitated with endogenous NPM (data not shown), our experiments nevertheless could not totally exclude an involvement of Sirt7, either independently or in association with Sirt1, in modulating NSR.

Our findings also suggest a novel mechanism by which Sirt1 can modulate p53 accumulation independent of lysine deacetylation. It has been observed that silencing of Sirt1 expression results in an increase in p53 protein accumulation (Ford et al., 2005). However, in vivo data indicate that p53 protein stability is uncoupled from its acetylation status (Cheng et al., 2003). Moreover, several recent studies have shown that Sirt2 can also deacetylate K382 of p53 (van Leeuwen et al., 2013), but inhibition of Sirt2 cannot induce p53 accumulation like Sirt1 (McCarthy et al., 2013). Based on the observations that nucleolar disruption (stress) has a pivotal role in governing p53 accumulation (Boyd et al., 2011; Rubbi & Milner, 2003), we suggest that Sirt1‐mediated inhibition of NSR may represent an additional, deacetylation‐independent mechanism for limiting p53 stabilization. Hence, our data provide a plausible explanation to the seemingly contradictory findings from previous studies. Nonetheless, it should be noted that this effect of Sirt1 may not necessarily be a beneficial process. For example, recent studies have indicated that Sirt1‐dependent repression of p53 activation may compromise the therapeutic effects of chemotherapy in leukemic cells (Li et al., 2012; Sasca et al., 2014).

Supporting our current results, several recent studies have also pointed to a crucial role of Sirt1 in orchestrating nucleolar events (Voit, Seiler, & Grummt, 2015). In particular, it has been shown that Sirt1 can interact (maybe indirectly) with another nucleolar protein, nucleomethylin, and formation of this protein complex is involved in modulating ribosomal biogenesis and cellular senescence in response to changes in energy supply (Murayama et al., 2008; Yang et al., 2013; Yang, Song, Chen, Soliman, & Chen, 2015). To our knowledge, our study is the first which reports that Sirt1 is implicated in regulating NSR. In addition to the activation of the p53 pathway, NSR may also signal to other pathways implicated in regulating cell cycle and apoptosis (James et al., 2014). Interestingly, we and other groups have observed in different cells that induction of NSR by selective inhibition of RNA polymerase I triggers activation of the ATR pathway, possibly independent of DNA strand breaks (Ma & Pederson, 2013; Quin et al., 2016; Ye et al., 2017). Hence, it is plausible that the nucleolus serves as a signaling hub, while Sirt1 may intervene with multiple pathways by regulating the occurrence of NSR.

In summary, our results suggest that Sirt1 is an endogenous suppressor of NSR, and this effect is partially independent of its deacetylase activity, while the physical interaction between Sirt1 and NPM appears to be required for NSR inhibition. Based on the reported prerequisite role of NSR in stress‐induced p53 protein accumulation, Sirt1‐mediated NSR inhibition may represent a novel mechanism by which Sirt1 modulates intracellular p53 accumulation independent of lysine deacetylation.

## 4 EXPERIMENTAL PROCEDURES

A detailed Experimental Procedures section can be found in Supporting information Methods.

### 4.1 SILAC and mass spectrometry analysis

Stable isotope labeling by amino acids in cell culture (SILAC) technique was used to identify Sirt1‐binding partners (Gruhler & Kratchmarova, 2008).

### 4.2 In vitro deacetylation assay

In vitro deacetylation assay was performed as described by Daitoku et al. (2004). The acetylation status of different lysine residues was characterized by mass spectrometry.

### 4.3 Live cell imaging

HeLa cells expressing eGFP‐NPM were continuously monitored with a spinning‐disk confocal live cell imaging system (Cell Observer SD; Zeiss) equipped with a top cage incubator. Fluorescent photographs were taken every 15 min for a total period of 24 hr, with a laser excitation at 490 nm.

### 4.4 Statistics

Data are presented as mean ± standard error of the mean (*SEM*). Data analysis was performed using unpaired *t* test or one‐way analysis of variance (ANOVA) followed by post hoc Tukey's test as appropriate. *p* < 0.05 was considered as statistically significant.

## CONFLICT OF INTEREST

None declared.

## AUTHOR CONTRIBUTIONS

Xiaolei Bi and Qing Ye performed the experiments, involved in data acquisition and analysis, and drafted the manuscript. Daoyuan Li and Zhe Wang involved in data acquisition and analysis. Qisheng Peng designed the experiment and involved in data analysis. Xiao Wu performed the experiments and involved in data acquisition. Yun Zhang involved in data analysis and interpretation. Qunye Zhang designed the experiment and involved in data acquisition, analysis, and interpretation. Fan Jiang conceived the study; designed the experiment; involved in data acquisition, analysis, and interpretation; and wrote and revised the manuscript.
