# Supplementary material for: Inhibition of nucleolar stress response by Sirt1: A potential mechanism of acetylation‐independent regulation of p53 accumulation
Source: Aging Cell. 2019 Jan 8;18(2):e12900. doi: 10.1111/acel.12900 (PMC6413664; doi:10.1111/acel.12900)
Supplement: Supplementary file 1 [file ACEL-18-e12900-s001.pdf]

Supplemental Figure S1

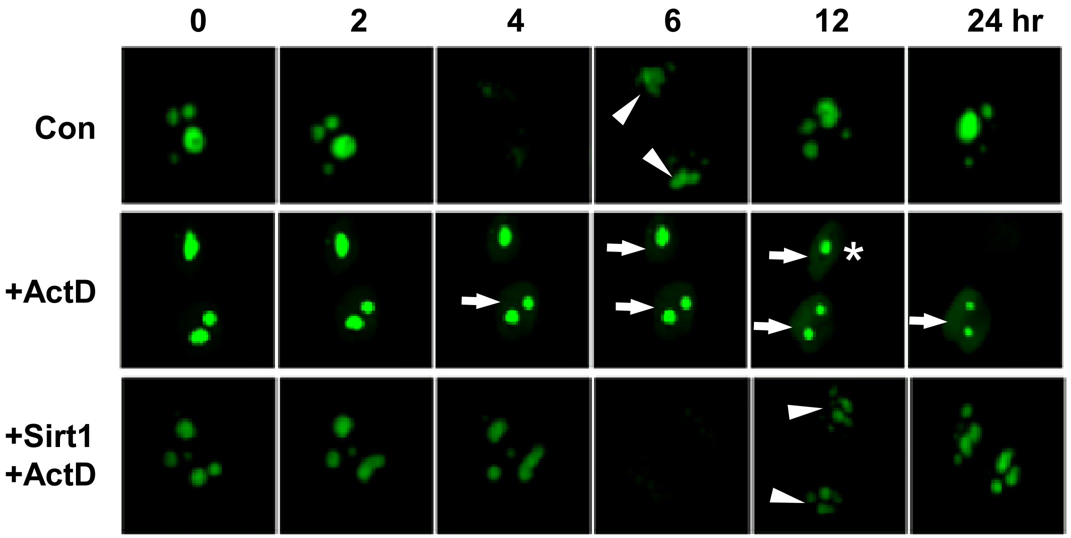

Supplemental Figure S1. Sirt1-dependent inhibition of ActD-induced NSR revealed by live cell imaging. In normal cells, transfected eGFP-NPM was exclusively expressed in the nucleolar compartment. ActD-induced NSR was demonstrated by the diffusion of eGFP signal from nucleoli to the nucleoplasm (arrows). Some cells entered into cell cycle; the nucleoli disassembled before cell division and reassembled in newly formed daughter cells (indicated by arrowheads). Cell division events were also observed in ActD-treated cells with a lower frequency (asterisk); however, the reassembling of nucleoli did not occur as in control and Sirt1-overexpressing cells.
