# Supplementary material for: Inhibition of nucleolar stress response by Sirt1: A potential mechanism of acetylation‐independent regulation of p53 accumulation
Source: Aging Cell. 2019 Jan 8;18(2):e12900. doi: 10.1111/acel.12900 (PMC6413664; doi:10.1111/acel.12900)
Supplement: Supplementary file 2 [file ACEL-18-e12900-s002.pdf]

Supplemental Figure S2

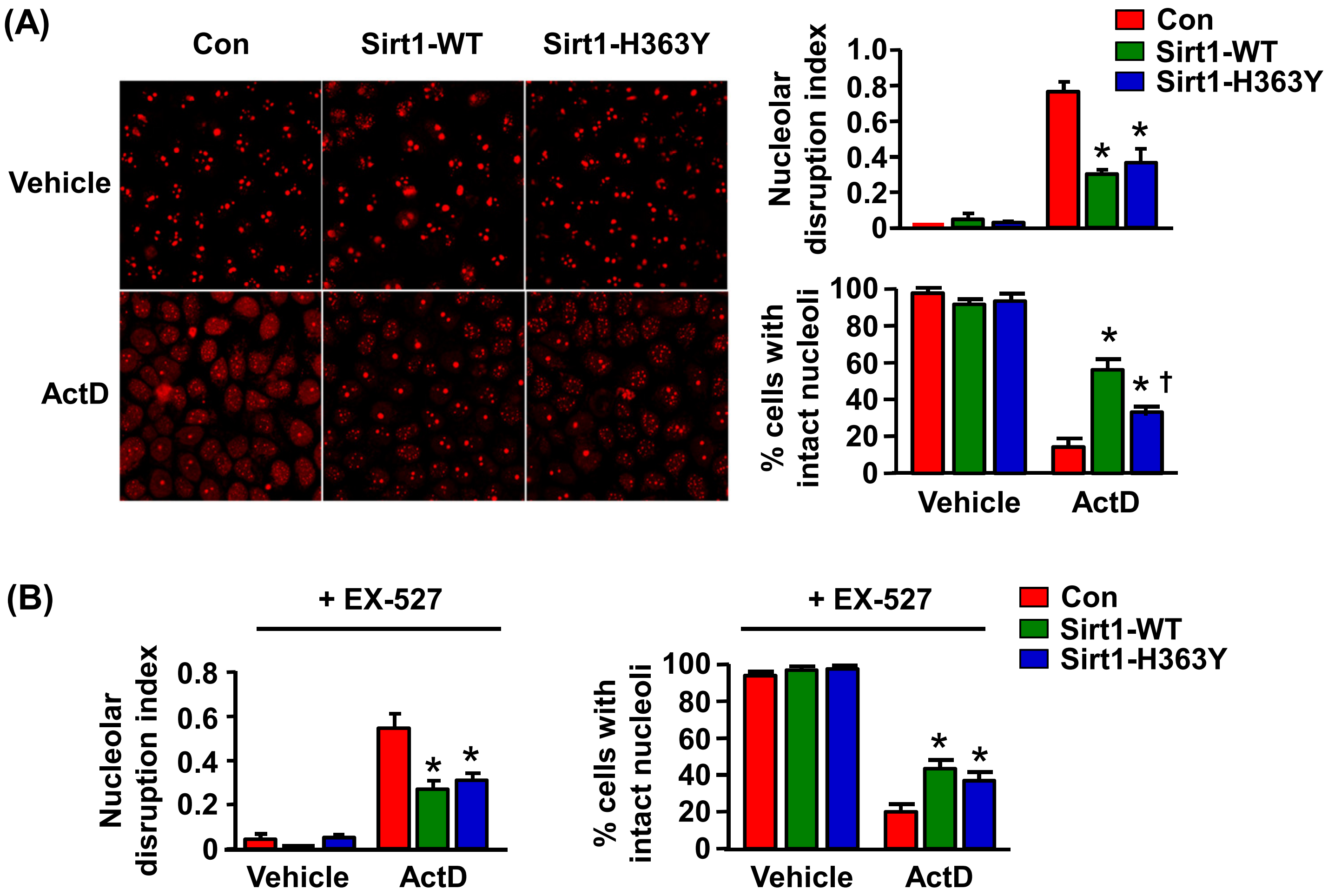

Supplemental Figure S2. Deacetylase activity-independent effect of Sirt1 on ActD-induced NSR. (A) Immunofluorescence images of NPM and quantitative data showing that overexpression of wild type (WT) and the enzymatically dead H363Y mutant of Sirt1 both repressed ActD (5 nM)-induced NSR. (B) The same experiments were repeated in cells pretreated with the Sirt1 inhibitor EX-527 (10 μM). Data are mean ± SEM. * $P < 0.05$ *versus* Con, † $P < 0.05$ *versus* WT, one-way ANOVA, $n = 5$.
