# Supplementary material for: Inhibition of nucleolar stress response by Sirt1: A potential mechanism of acetylation‐independent regulation of p53 accumulation
Source: Aging Cell. 2019 Jan 8;18(2):e12900. doi: 10.1111/acel.12900 (PMC6413664; doi:10.1111/acel.12900)
Supplement: Supplementary file 3 [file ACEL-18-e12900-s003.pdf]

Supplemental Table S1. Sirt1 binding proteins detected by SILAC-based proteomic analysis

Mascot Distiller Quantitation Report

Sample: 20130313IPSILAC
Project file: file:///D:/daoyuanli/SILAC/20130313IPSILAC.rov
Timestamp: Friday, March 15, 2013 11:04:30
Raw file 1: D:\daoyuanli\LTQ-Orbitrap\20130313IPSILAC.raw
Instrument method 1: D:\Chi Lianli\Li Daoyuan\2013\IP_SILAC\IP SILAC.meth
Quantitation method: SILAC R+4 (15N) LDY
1 component SILAC, 99%
Mascot search results: Open in new window

Hit	Member	Accession	Score	Mass	L/H	SD (geo)	#	Description
1	1	ROA2_HUMAN	2506	37407	**1.319**	**1.089**	**12**	Heterogeneous nuclear ribonucleoproteins A2/B1 OS=Homo sapiens GN=HNRNPA2B1 PE=1 SV=2
1	2	ROA1_HUMAN	1867	38823	**1.781**	**1.035**	**12**	Heterogeneous nuclear ribonucleoprotein A1 OS=Homo sapiens GN=HNRNPA1 PE=1 SV=5
1	3	ROA3_HUMAN	608	39686	**1.618**	**1.027**	**4**	Heterogeneous nuclear ribonucleoprotein A3 OS=Homo sapiens GN=HNRNPA3 PE=1 SV=2
1	4	ROA0_HUMAN	134	30886				Heterogeneous nuclear ribonucleoprotein A0 OS=Homo sapiens GN=HNRNPA0 PE=1 SV=1
2		DHX9_HUMAN	1511	140869	**1.462**	**1.068**	**16**	ATP-dependent RNA helicase A OS=Homo sapiens GN=DHX9 PE=1 SV=4
3	1	K1C10_HUMAN	1161	58792	**95.60**	**3.630**	**20**	Keratin, type I cytoskeletal 10 OS=Homo sapiens GN=KRT10 PE=1 SV=6
3	2	K1C14_HUMAN	188	51529	**124.4**	**3.894**	**4**	Keratin, type I cytoskeletal 14 OS=Homo sapiens GN=KRT14 PE=1 SV=4
3	3	K1C16_HUMAN	141	51236	**99.44**	**3.249**	**5**	Keratin, type I cytoskeletal 16 OS=Homo sapiens GN=KRT16 PE=1 SV=4
3	4	K1C18_HUMAN	55	48029	199.4	16.70	3	Keratin, type I cytoskeletal 18 OS=Homo sapiens GN=KRT18 PE=1 SV=2
4		HNRPK_HUMAN	1066	51088	**1.532**	**1.075**	**9**	Heterogeneous nuclear ribonucleoprotein K OS=Homo sapiens GN=HNRNPK PE=1 SV=1
5	1	PABP1_HUMAN	915	70797	**1.667**	**1.074**	**8**	Polyadenylate-binding protein 1 OS=Homo sapiens GN=PABPC1 PE=1 SV=2
5	2	PABP4_HUMAN	491	70738	**1.677**	**1.105**	**5**	Polyadenylate-binding protein 4 OS=Homo sapiens GN=PABPC4 PE=1 SV=1
6	1	K2C1_HUMAN	868	65999	**226.6**	**4.826**	**13**	Keratin, type II cytoskeletal 1 OS=Homo sapiens GN=KRT1

								PE=1 SV=6
6	2	K22E_HUMAN	433	65521	**160.9**	**4.039**	**10**	Keratin, type II cytoskeletal 2 epidermal OS=Homo sapiens GN=KRT2 PE=1 SV=2
6	3	K2C5_HUMAN	126	62488	**155.1**	**3.702**	**6**	Keratin, type II cytoskeletal 5 OS=Homo sapiens GN=KRT5 PE=1 SV=3
6	4	K2C8_HUMAN	105	53799	**247.1**	**4.483**	**3**	Keratin, type II cytoskeletal 8 OS=Homo sapiens GN=KRT8 PE=1 SV=7
6	5	K2C6A_HUMAN	86	60152	**247.1**	**4.105**	**5**	Keratin, type II cytoskeletal 6A OS=Homo sapiens GN=KRT6A PE=1 SV=3
7		HNRPU_HUMAN	804	90707	**1.479**	**1.114**	**8**	Heterogeneous nuclear ribonucleoprotein U OS=Homo sapiens GN=HNRNPU PE=1 SV=6
8		K1C9_HUMAN	748	62027	613.3	254.5	11	Keratin, type I cytoskeletal 9 OS=Homo sapiens GN=KRT9 PE=1 SV=3
9		SFPQ_HUMAN	701	76341	**1.569**	**1.120**	**12**	Splicing factor, proline- and glutamine-rich OS=Homo sapiens GN=SFPQ PE=1 SV=2
10	1	DDX5_HUMAN	694	69105	**1.846**	**1.150**	**10**	Probable ATP-dependent RNA helicase DDX5 OS=Homo sapiens GN=DDX5 PE=1 SV=1
10	2	DDX3X_HUMAN	523	73417	**1.754**	**1.057**	**5**	ATP-dependent RNA helicase DDX3X OS=Homo sapiens GN=DDX3X PE=1 SV=3
10	3	DDX17_HUMAN	492	80457	**1.754**	**1.193**	**3**	Probable ATP-dependent RNA helicase DDX17 OS=Homo sapiens GN=DDX17 PE=1 SV=2
11	1	SMRC2_HUMAN	682	132980				SWI/SNF complex subunit SMARCC2 OS=Homo sapiens GN=SMARCC2 PE=1 SV=1
11	2	SMRC1_HUMAN	568	122994				SWI/SNF complex subunit SMARCC1 OS=Homo sapiens GN=SMARCC1 PE=1 SV=3
12	1	ACTB_HUMAN	664	41710	**1.416**	**1.046**	**8**	Actin, cytoplasmic 1 OS=Homo sapiens GN=ACTB PE=1 SV=1
12	2	ACTC_HUMAN	299	41992	**1.421**	**1.051**	**4**	Actin, alpha cardiac muscle 1 OS=Homo sapiens GN=ACTC1 PE=1 SV=1
13	1	HNRPR_HUMAN	654	71095	**1.421**	**1.015**	**3**	Heterogeneous nuclear ribonucleoprotein R OS=Homo sapiens GN=HNRNPR PE=1 SV=1
13	2	HNRPQ_HUMAN	329	69739				Heterogeneous nuclear ribonucleoprotein Q OS=Homo sapiens GN=SYNCRIP PE=1 SV=2
14	1	FUS_HUMAN	642	53541	**1.956**	**1.099**	**5**	RNA-binding protein FUS OS=Homo sapiens GN=FUS PE=1 SV=1
14	2	RBP56_HUMAN	249	62016				TATA-binding protein-associated factor 2N OS=Homo

								sapiens GN=TAF15 PE=1 SV=1
15		HNRH3_HUMAN	629	37015	**1.721**	**1.067**	**4**	Heterogeneous nuclear ribonucleoprotein H3 OS=Homo sapiens GN=HNRNPH3 PE=1 SV=2
16		TFG_HUMAN	609	43485	**1.708**	**1.085**	**11**	Protein TFG OS=Homo sapiens GN=TFG PE=1 SV=2
17		HNRPC_HUMAN	589	33721				Heterogeneous nuclear ribonucleoproteins C1/C2 OS=Homo sapiens GN=HNRNPC PE=1 SV=4
18		DDX1_HUMAN	585	82380	**1.450**	**1.037**	**2**	ATP-dependent RNA helicase DDX1 OS=Homo sapiens GN=DDX1 PE=1 SV=2
19		HNRPL_HUMAN	530	64092	**1.699**	**1.017**	**2**	Heterogeneous nuclear ribonucleoprotein L OS=Homo sapiens GN=HNRNPL PE=1 SV=2
20	1	HNRH1_HUMAN	525	49322	**1.660**	**1.074**	**5**	Heterogeneous nuclear ribonucleoprotein H OS=Homo sapiens GN=HNRNPH1 PE=1 SV=4
20	2	HNRPF_HUMAN	368	45739	1.617	1.136	2	Heterogeneous nuclear ribonucleoprotein F OS=Homo sapiens GN=HNRNPF PE=1 SV=3
20	3	HNRH2_HUMAN	360	49356	**1.660**	**1.083**	**3**	Heterogeneous nuclear ribonucleoprotein H2 OS=Homo sapiens GN=HNRNPH2 PE=1 SV=1
21		ILF3_HUMAN	508	95279	1.510	1.081	2	Interleukin enhancer-binding factor 3 OS=Homo sapiens GN=ILF3 PE=1 SV=3
22		FA98A_HUMAN	490	55494	**1.447**	**1.069**	**3**	Protein FAM98A OS=Homo sapiens GN=FAM98A PE=1 SV=1
23		TBA1B_HUMAN	483	50203				Tubulin alpha-1B chain OS=Homo sapiens GN=TUBA1B PE=1 SV=1
24	1	TBB5_HUMAN	477	49727	**1.605**	**1.052**	**2**	Tubulin beta chain OS=Homo sapiens GN=TUBB PE=1 SV=2
24	2	TBB4B_HUMAN	420	49887				Tubulin beta-4B chain OS=Homo sapiens GN=TUBB4B PE=1 SV=1
25		SAHH_HUMAN	393	47753				Adenosylhomocysteinase OS=Homo sapiens GN=AHCY PE=1 SV=4
26		CCAR1_HUMAN	392	133066				Cell division cycle and apoptosis regulator protein 1 OS=Homo sapiens GN=CCAR1 PE=1 SV=2
27		RTCB_HUMAN	387	55175	1.174	1.090	2	tRNA-splicing ligase RtcB homolog OS=Homo sapiens GN=C22orf28 PE=1 SV=1
28		EF1D_HUMAN	386	31167	146.4	478.1	2	Elongation factor 1-delta OS=Homo sapiens GN=EEF1D PE=1 SV=5

29	1	EF1A2_HUMAN	384	50510	**1.568**	**1.123**	**3**	Elongation factor 1-alpha 2 OS=Homo sapiens GN=EEF1A2 PE=1 SV=1
29	2	EF1A1_HUMAN	340	50177	1.676	1.059	2	Elongation factor 1-alpha 1 OS=Homo sapiens GN=EEF1A1 PE=1 SV=1
30		MVP_HUMAN	371	99266	3.490	8.993	3	Major vault protein OS=Homo sapiens GN=MVP PE=1 SV=4
31		HNRL1_HUMAN	370	95943	**2.004**	**1.133**	**5**	Heterogeneous nuclear ribonucleoprotein U-like protein 1 OS=Homo sapiens GN=HNRNPUL1 PE=1 SV=2
32	1	G3BP1_HUMAN	367	52280				Ras GTPase-activating protein-binding protein 1 OS=Homo sapiens GN=G3BP1 PE=1 SV=1
32	2	G3BP2_HUMAN	105	54088				Ras GTPase-activating protein-binding protein 2 OS=Homo sapiens GN=G3BP2 PE=1 SV=2
33		EF1G_HUMAN	365	50175	**1.520**	**1.045**	**5**	Elongation factor 1-gamma OS=Homo sapiens GN=EEF1G PE=1 SV=3
34		CH60_HUMAN	358	61104				60 kDa heat shock protein, mitochondrial OS=Homo sapiens GN=HSPD1 PE=1 SV=2
35		ARI1A_HUMAN	352	242239				AT-rich interactive domain-containing protein 1A OS=Homo sapiens GN=ARID1A PE=1 SV=3
36		ILF2_HUMAN	350	43127	**1.418**	**1.015**	**2**	Interleukin enhancer-binding factor 2 OS=Homo sapiens GN=ILF2 PE=1 SV=2
37		NONO_HUMAN	342	54381	**1.645**	**1.107**	**5**	Non-POU domain-containing octamer-binding protein OS=Homo sapiens GN=NONO PE=1 SV=4
38		CAPR1_HUMAN	331	78454				Caprin-1 OS=Homo sapiens GN=CAPRIN1 PE=1 SV=2
39		HNRPM_HUMAN	329	77680	0.6530	2198	2	Heterogeneous nuclear ribonucleoprotein M OS=Homo sapiens GN=HNRNPM PE=1 SV=3
40	1	H2B1C_HUMAN	320	13898	**1.454**	**1.086**	**3**	Histone H2B type 1-C/E/F/G/I OS=Homo sapiens GN=HIST1H2BC PE=1 SV=4
40	2	H2B1B_HUMAN	310	13942	**1.454**	**1.086**	**3**	Histone H2B type 1-B OS=Homo sapiens GN=HIST1H2BB PE=1 SV=2
41		CN166_HUMAN	313	28115	**1.509**	**1.134**	**3**	UPF0568 protein C14orf166 OS=Homo sapiens GN=C14orf166 PE=1 SV=1
42		TERA_HUMAN	298	89266				Transitional endoplasmic reticulum ATPase OS=Homo sapiens GN=VCP PE=1 SV=4
43		FLNA_HUMAN	290	280935				Filamin-A OS=Homo sapiens GN=FLNA PE=1 SV=4

44	1	HSP7C_HUMAN	285	70854				Heat shock cognate 71 kDa protein OS=Homo sapiens GN=HSPA8 PE=1 SV=1
44	2	GRP78_HUMAN	241	72400				78 kDa glucose-regulated protein OS=Homo sapiens GN=HSPA5 PE=1 SV=2
44	3	HSP71_HUMAN	137	70009				Heat shock 70 kDa protein 1A/1B OS=Homo sapiens GN=HSPA1A PE=1 SV=5
45	1	H2A2A_HUMAN	284	14087	**1.407**	**1.121**	**5**	Histone H2A type 2-A OS=Homo sapiens GN=HIST2H2AA3 PE=1 SV=3
45	2	H2A1B_HUMAN	265	14127	**1.407**	**1.115**	**5**	Histone H2A type 1-B/E OS=Homo sapiens GN=HIST1H2AB PE=1 SV=2
45	3	H2AV_HUMAN	84	13501	**1.372**	**1.079**	**3**	Histone H2A.V OS=Homo sapiens GN=H2AFV PE=1 SV=3
46		RS2_HUMAN	278	31400				40S ribosomal protein S2 OS=Homo sapiens GN=RPS2 PE=1 SV=2
47	1	AGO2_HUMAN	243	97373				Protein argonaute-2 OS=Homo sapiens GN=EIF2C2 PE=1 SV=3
47	2	AGO3_HUMAN	50	97545				Protein argonaute-3 OS=Homo sapiens GN=EIF2C3 PE=1 SV=2
48		RS15_HUMAN	236	17089				40S ribosomal protein S15 OS=Homo sapiens GN=RPS15 PE=1 SV=2
49	1	SMCA4_HUMAN	232	184530				Transcription activator BRG1 OS=Homo sapiens GN=SMARCA4 PE=1 SV=2
49	2	SMCA2_HUMAN	190	181166				Probable global transcription activator SNF2L2 OS=Homo sapiens GN=SMARCA2 PE=1 SV=2
50		RU17_HUMAN	231	51526	**1.583**	**1.117**	**4**	U1 small nuclear ribonucleoprotein 70 kDa OS=Homo sapiens GN=SNRNP70 PE=1 SV=2
51		SNRPA_HUMAN	225	31311				U1 small nuclear ribonucleoprotein A OS=Homo sapiens GN=SNRPA PE=1 SV=3
52		MYH9_HUMAN	218	226882				Myosin-9 OS=Homo sapiens GN=MYH9 PE=1 SV=4
53		SMD1_HUMAN	217	13337	1.678	1.089	2	Small nuclear ribonucleoprotein Sm D1 OS=Homo sapiens GN=SNRPD1 PE=1 SV=1
54	1	YBOX1_HUMAN	216	36058				Nuclease-sensitive element-binding protein 1 OS=Homo sapiens GN=YBX1 PE=1 SV=3
54	2	DBPA_HUMAN	150	40230				DNA-binding protein A OS=Homo sapiens GN=CSDA PE=1 SV=4
								60S ribosomal protein L6

55		RL6_HUMAN	216	32795				OS=Homo sapiens GN=RPL6 PE=1 SV=3
56		SMD2_HUMAN	213	13546	1.627	1.112	2	Small nuclear ribonucleoprotein Sm D2 OS=Homo sapiens GN=SNRPD2 PE=1 SV=1
57	1	HNRDL_HUMAN	209	46541	1.610	1.090	2	Heterogeneous nuclear ribonucleoprotein D-like OS=Homo sapiens GN=HNRPDL PE=1 SV=3
57	2	HNRPD_HUMAN	204	38466				Heterogeneous nuclear ribonucleoprotein D0 OS=Homo sapiens GN=HNRNPD PE=1 SV=1
57	3	ROAA_HUMAN	203	36262				Heterogeneous nuclear ribonucleoprotein A/B OS=Homo sapiens GN=HNRNPAB PE=1 SV=2
58		MATR3_HUMAN	201	94565				Matrin-3 OS=Homo sapiens GN=MATR3 PE=1 SV=2
59		RL9_HUMAN	199	21914				60S ribosomal protein L9 OS=Homo sapiens GN=RPL9 PE=1 SV=1
60		CLH1_HUMAN	199	191820				Clathrin heavy chain 1 OS=Homo sapiens GN=CLTC PE=1 SV=5
61		EF1B_HUMAN	191	24768				Elongation factor 1-beta OS=Homo sapiens GN=EEF1B2 PE=1 SV=3
62		RS7_HUMAN	191	22169				40S ribosomal protein S7 OS=Homo sapiens GN=RPS7 PE=1 SV=1
63		PTBP1_HUMAN	189	57257				Polypyrimidine tract-binding protein 1 OS=Homo sapiens GN=PTBP1 PE=1 SV=1
64		THOC4_HUMAN	186	26872				THO complex subunit 4 OS=Homo sapiens GN=ALYREF PE=1 SV=3
65		H4_HUMAN	181	11360	**1.370**	**1.126**	**4**	Histone H4 OS=Homo sapiens GN=HIST1H4A PE=1 SV=2
66		EWS_HUMAN	180	68615				RNA-binding protein EWS OS=Homo sapiens GN=EWSR1 PE=1 SV=1
67		ELAV1_HUMAN	180	36149	1.790	1.163	2	ELAV-like protein 1 OS=Homo sapiens GN=ELAVL1 PE=1 SV=2
68		SAFB1_HUMAN	173	102580				Scaffold attachment factor B1 OS=Homo sapiens GN=SAFB PE=1 SV=4
69		RS3A_HUMAN	170	29982				40S ribosomal protein S3a OS=Homo sapiens GN=RPS3A PE=1 SV=2
70	1	SRSF1_HUMAN	162	27728	1.380	39.37	3	Serine/arginine-rich splicing factor 1 OS=Homo sapiens GN=SRSF1 PE=1 SV=2
70	2	SRSF9_HUMAN	40	25526				Serine/arginine-rich splicing factor 9 OS=Homo sapiens GN=SRSF9 PE=1 SV=1

71		RL7_HUMAN	154	29315					60S ribosomal protein L7 OS=Homo sapiens GN=RPL7 PE=1 SV=1
72		SC23A_HUMAN	153	86277					Protein transport protein Sec23A OS=Homo sapiens GN=SEC23A PE=1 SV=2
73		RL12_HUMAN	150	17847					60S ribosomal protein L12 OS=Homo sapiens GN=RPL12 PE=1 SV=1
74		NPM_HUMAN	149	32587					Nucleophosmin OS=Homo sapiens GN=NPM1 PE=1 SV=2
75		RS8_HUMAN	147	24282					40S ribosomal protein S8 OS=Homo sapiens GN=RPS8 PE=1 SV=2
76		CBX1_HUMAN	145	21432					Chromobox protein homolog 1 OS=Homo sapiens GN=CBX1 PE=1 SV=1
77		U520_HUMAN	142	244843					U5 small nuclear ribonucleoprotein 200 kDa helicase OS=Homo sapiens GN=SNRNP200 PE=1 SV=2
78		RL3_HUMAN	142	46199					60S ribosomal protein L3 OS=Homo sapiens GN=RPL3 PE=1 SV=2
79		DDX6_HUMAN	141	54482					Probable ATP-dependent RNA helicase DDX6 OS=Homo sapiens GN=DDX6 PE=1 SV=2
80		RS14_HUMAN	140	16263					40S ribosomal protein S14 OS=Homo sapiens GN=RPS14 PE=1 SV=3
81		ADT2_HUMAN	140	32899					ADP/ATP translocase 2 OS=Homo sapiens GN=SLC25A5 PE=1 SV=7
82		LARP1_HUMAN	138	123434					La-related protein 1 OS=Homo sapiens GN=LARP1 PE=1 SV=2
83		ATX2L_HUMAN	135	113499					Ataxin-2-like protein OS=Homo sapiens GN=ATXN2L PE=1 SV=2
84		RL7A_HUMAN	133	30069					60S ribosomal protein L7a OS=Homo sapiens GN=RPL7A PE=1 SV=2
85		RL18A_HUMAN	132	20749					60S ribosomal protein L18a OS=Homo sapiens GN=RPL18A PE=1 SV=2
86		RS16_HUMAN	131	16435					40S ribosomal protein S16 OS=Homo sapiens GN=RPS16 PE=1 SV=2
87		SNF5_HUMAN	130	44209					SWI/SNF-related matrix-associated actin-dependent regulator of chromatin subfamily B member 1 OS=Homo sapiens GN=SMARCB1 PE=1 SV=2
88		RUXG_HUMAN	128	8506	1.563	111.5	3		Small nuclear ribonucleoprotein G OS=Homo sapiens GN=SNRPG PE=1 SV=1

89		PRP8_HUMAN	126	274021				Pre-mRNA-processing-splicing factor 8 OS=Homo sapiens GN=PRPF8 PE=1 SV=2
90		RUXE_HUMAN	125	10825				Small nuclear ribonucleoprotein E OS=Homo sapiens GN=SNRPE PE=1 SV=1
91	1	SMRD1_HUMAN	124	58196				SWI/SNF-related matrix-associated actin-dependent regulator of chromatin subfamily D member 1 OS=Homo sapiens GN=SMARCD1 PE=1 SV=2
91	2	SMRD2_HUMAN	114	58884				SWI/SNF-related matrix-associated actin-dependent regulator of chromatin subfamily D member 2 OS=Homo sapiens GN=SMARCD2 PE=1 SV=3
92		RS4X_HUMAN	123	29579				40S ribosomal protein S4, X isoform OS=Homo sapiens GN=RPS4X PE=1 SV=2
93		RLA2_HUMAN	121	11666				60S acidic ribosomal protein P2 OS=Homo sapiens GN=RPLP2 PE=1 SV=1
94		RU1C_HUMAN	121	17417				U1 small nuclear ribonucleoprotein C OS=Homo sapiens GN=SNRPC PE=1 SV=1
95		RL13_HUMAN	118	24247	1.854	1.090	2	60S ribosomal protein L13 OS=Homo sapiens GN=RPL13 PE=1 SV=4
96		RBM14_HUMAN	118	69612				RNA-binding protein 14 OS=Homo sapiens GN=RBM14 PE=1 SV=2
97		RL19_HUMAN	118	23451	**1.736**	**1.017**	**2**	60S ribosomal protein L19 OS=Homo sapiens GN=RPL19 PE=1 SV=1
98		ACTN4_HUMAN	117	104992				Alpha-actinin-4 OS=Homo sapiens GN=ACTN4 PE=1 SV=2
99		RBMX_HUMAN	111	42534				RNA-binding motif protein, X chromosome OS=Homo sapiens GN=RBMX PE=1 SV=3
100		RLA0_HUMAN	111	34304				60S acidic ribosomal protein P0 OS=Homo sapiens GN=RPLP0 PE=1 SV=1
101		F120A_HUMAN	111	122070				Constitutive coactivator of PPAR-gamma-like protein 1 OS=Homo sapiens GN=FAM120A PE=1 SV=2
102		RS6_HUMAN	109	28663				40S ribosomal protein S6 OS=Homo sapiens GN=RPS6 PE=1 SV=1
103	1	TRA2B_HUMAN	107	33901				Transformer-2 protein homolog beta OS=Homo sapiens GN=TRA2B PE=1 SV=1
103	2	TRA2A_HUMAN	73	32908				Transformer-2 protein homolog alpha OS=Homo sapiens

								GN=TRA2A PE=1 SV=1
104	1	H32_HUMAN	107	15450				Histone H3.2 OS=Homo sapiens GN=HIST2H3A PE=1 SV=3
104	2	H31_HUMAN	59	15466				Histone H3.1 OS=Homo sapiens GN=HIST1H3A PE=1 SV=2
104	3	H33_HUMAN	27	15390				Histone H3.3 OS=Homo sapiens GN=H3F3A PE=1 SV=2
105		RSMB_HUMAN	106	24673	**1.567**	**1.090**	**4**	Small nuclear ribonucleoprotein-associated proteins B and B' OS=Homo sapiens GN=SNRPB PE=1 SV=2
106	1	SRSF7_HUMAN	106	27350				Serine/arginine-rich splicing factor 7 OS=Homo sapiens GN=SRSF7 PE=1 SV=1
106	2	SRSF3_HUMAN	93	19477				Serine/arginine-rich splicing factor 3 OS=Homo sapiens GN=SRSF3 PE=1 SV=1
107		RALY_HUMAN	106	32527				RNA-binding protein Raly OS=Homo sapiens GN=RALY PE=1 SV=1
108		RL22_HUMAN	105	14802	1.712	1.093	2	60S ribosomal protein L22 OS=Homo sapiens GN=RPL22 PE=1 SV=2
109		RL23A_HUMAN	104	17728				60S ribosomal protein L23a OS=Homo sapiens GN=RPL23A PE=1 SV=1
110		ACL6A_HUMAN	104	47506				Actin-like protein 6A OS=Homo sapiens GN=ACTL6A PE=1 SV=1
111		RL15_HUMAN	104	24131				60S ribosomal protein L15 OS=Homo sapiens GN=RPL15 PE=1 SV=2
112		RS19_HUMAN	100	16098				40S ribosomal protein S19 OS=Homo sapiens GN=RPS19 PE=1 SV=2
113	1	HS90B_HUMAN	98	83212				Heat shock protein HSP 90-beta OS=Homo sapiens GN=HSP90AB1 PE=1 SV=4
113	2	HS902_HUMAN	63	39368				Putative heat shock protein HSP 90-alpha A2 OS=Homo sapiens GN=HSP90AA2 PE=1 SV=2
114		HNRL2_HUMAN	97	85291				Heterogeneous nuclear ribonucleoprotein U-like protein 2 OS=Homo sapiens GN=HNRNPUL2 PE=1 SV=1
115		SMD3_HUMAN	97	13955				Small nuclear ribonucleoprotein Sm D3 OS=Homo sapiens GN=SNRPD3 PE=1 SV=1
116		SF3B1_HUMAN	95	146053				Splicing factor 3B subunit 1 OS=Homo sapiens GN=SF3B1 PE=1 SV=3
117		RL17_HUMAN	95	21443				60S ribosomal protein L17 OS=Homo sapiens GN=RPL17

								PE=1 SV=3
118		RL8_HUMAN	94	28007				60S ribosomal protein L8 OS=Homo sapiens GN=RPL8 PE=1 SV=2
119	1	IF2B1_HUMAN	93	63557				Insulin-like growth factor 2 mRNA-binding protein 1 OS=Homo sapiens GN=IGF2BP1 PE=1 SV=2
119	2	IF2B3_HUMAN	39	63769				Insulin-like growth factor 2 mRNA-binding protein 3 OS=Homo sapiens GN=IGF2BP3 PE=1 SV=2
120		RO52_HUMAN	93	54255				E3 ubiquitin-protein ligase TRIM21 OS=Homo sapiens GN=TRIM21 PE=1 SV=1
121		SMCE1_HUMAN	93	46716				SWI/SNF-related matrix-associated actin-dependent regulator of chromatin subfamily E member 1 OS=Homo sapiens GN=SMARCE1 PE=1 SV=2
122		RS25_HUMAN	92	13758				40S ribosomal protein S25 OS=Homo sapiens GN=RPS25 PE=1 SV=1
123		RT29_HUMAN	92	45621				28S ribosomal protein S29, mitochondrial OS=Homo sapiens GN=DAP3 PE=1 SV=1
124		RS3_HUMAN	91	26743				40S ribosomal protein S3 OS=Homo sapiens GN=RPS3 PE=1 SV=2
125		RS18_HUMAN	90	17708				40S ribosomal protein S18 OS=Homo sapiens GN=RPS18 PE=1 SV=3
126		RS23_HUMAN	90	15846				40S ribosomal protein S23 OS=Homo sapiens GN=RPS23 PE=1 SV=3
127		SF3B2_HUMAN	89	100364				Splicing factor 3B subunit 2 OS=Homo sapiens GN=SF3B2 PE=1 SV=2
128		CC124_HUMAN	89	25820				Coiled-coil domain-containing protein 124 OS=Homo sapiens GN=CCDC124 PE=1 SV=1
129		NUFP2_HUMAN	88	76139				Nuclear fragile X mental retardation-interacting protein 2 OS=Homo sapiens GN=NUFIP2 PE=1 SV=1
130		RS5_HUMAN	86	22930				40S ribosomal protein S5 OS=Homo sapiens GN=RPS5 PE=1 SV=4
131		RS24_HUMAN	85	15465				40S ribosomal protein S24 OS=Homo sapiens GN=RPS24 PE=1 SV=1
132		NUCL_HUMAN	84	76568				Nucleolin OS=Homo sapiens GN=NCL PE=1 SV=3
133		RL30_HUMAN	83	12776				60S ribosomal protein L30 OS=Homo sapiens GN=RPL30 PE=1 SV=2

134		RL4_HUMAN	81	47831					60S ribosomal protein L4 OS=Homo sapiens GN=RPL4 PE=1 SV=5
135		RS17L_HUMAN	80	15592					40S ribosomal protein S17-like OS=Homo sapiens GN=RPS17L PE=3 SV=1
136		WIPI3_HUMAN	80	38141					WD repeat domain phosphoinositide-interacting protein 3 OS=Homo sapiens GN=WDR45L PE=2 SV=2
137		RS20_HUMAN	80	13364					40S ribosomal protein S20 OS=Homo sapiens GN=RPS20 PE=1 SV=1
138		DHX15_HUMAN	79	90875					Putative pre-mRNA-splicing factor ATP-dependent RNA helicase DHX15 OS=Homo sapiens GN=DHX15 PE=1 SV=2
139		SSXT_HUMAN	77	45935					Protein SSXT OS=Homo sapiens GN=SS18 PE=1 SV=3
140		BCL7C_HUMAN	77	23454					B-cell CLL/lymphoma 7 protein family member C OS=Homo sapiens GN=BCL7C PE=1 SV=3
141		RL14_HUMAN	76	23417					60S ribosomal protein L14 OS=Homo sapiens GN=RPL14 PE=1 SV=4
142		RS9_HUMAN	76	22578					40S ribosomal protein S9 OS=Homo sapiens GN=RPS9 PE=1 SV=3
143		KHDR1_HUMAN	75	48333					KH domain-containing, RNA-binding, signal transduction-associated protein 1 OS=Homo sapiens GN=KHDRBS1 PE=1 SV=1
144		MYO1C_HUMAN	73	121965					Unconventional myosin-Ic OS=Homo sapiens GN=MYO1C PE=1 SV=4
145		SR140_HUMAN	72	118538					U2 snRNP-associated SURP motif-containing protein OS=Homo sapiens GN=U2SURP PE=1 SV=2
146		RL23_HUMAN	71	14900					60S ribosomal protein L23 OS=Homo sapiens GN=RPL23 PE=1 SV=1
147		RL10A_HUMAN	71	24859					60S ribosomal protein L10a OS=Homo sapiens GN=RPL10A PE=1 SV=2
148		NCBP1_HUMAN	69	91952					Nuclear cap-binding protein subunit 1 OS=Homo sapiens GN=NCBP1 PE=1 SV=1
149		RL32_HUMAN	68	15850					60S ribosomal protein L32 OS=Homo sapiens GN=RPL32 PE=1 SV=2
150		PM14_HUMAN	68	14611					Pre-mRNA branch site protein p14 OS=Homo sapiens GN=SF3B14 PE=1 SV=1
									60S ribosomal protein L31

151		RL31_HUMAN	67	14454				OS=Homo sapiens GN=RPL31 PE=1 SV=1
152		EIF3F_HUMAN	66	37588				Eukaryotic translation initiation factor 3 subunit F OS=Homo sapiens GN=EIF3F PE=1 SV=1
153		RT27_HUMAN	65	47653				28S ribosomal protein S27, mitochondrial OS=Homo sapiens GN=MRPS27 PE=1 SV=3
154		PRKDC_HUMAN	64	469645				DNA-dependent protein kinase catalytic subunit OS=Homo sapiens GN=PRKDC PE=1 SV=3
155		RS15A_HUMAN	63	14874				40S ribosomal protein S15a OS=Homo sapiens GN=RPS15A PE=1 SV=2
156		THIO_HUMAN	62	11730				Thioredoxin OS=Homo sapiens GN=TXN PE=1 SV=3
157		IGHG1_HUMAN	61	36083	**984.5**	**45.72**	**4**	Ig gamma-1 chain C region OS=Homo sapiens GN=IGHG1 PE=1 SV=1
158		DHX36_HUMAN	61	114947				Probable ATP-dependent RNA helicase DHX36 OS=Homo sapiens GN=DHX36 PE=1 SV=2
159		CPSM_HUMAN	59	165062				Carbamoyl-phosphate synthase [ammonia], mitochondrial OS=Homo sapiens GN=CPS1 PE=1 SV=2
160		TIA1_HUMAN	59	42936				Nucleolysin TIA-1 isoform p40 OS=Homo sapiens GN=TIA1 PE=1 SV=3
161		1433T_HUMAN	58	27791				14-3-3 protein theta OS=Homo sapiens GN=YWHAQ PE=1 SV=1
162		RSBNL_HUMAN	58	94990				Round spermatid basic protein 1-like protein OS=Homo sapiens GN=RSBN1L PE=1 SV=2
163		KBTB3_HUMAN	58	69422				Kelch repeat and BTB domain-containing protein 3 OS=Homo sapiens GN=KBTBD3 PE=2 SV=2
164		RL29_HUMAN	58	17801				60S ribosomal protein L29 OS=Homo sapiens GN=RPL29 PE=1 SV=2
165		E2AK2_HUMAN	57	62159				Interferon-induced, double-stranded RNA-activated protein kinase OS=Homo sapiens GN=EIF2AK2 PE=1 SV=2
166		RL27A_HUMAN	56	16551				60S ribosomal protein L27a OS=Homo sapiens GN=RPL27A PE=1 SV=2
167		RS13_HUMAN	56	17268				40S ribosomal protein S13 OS=Homo sapiens GN=RPS13 PE=1 SV=2
								ATPase family AAA domain-containing protein 3C

168		ATD3C_HUMAN	55	46350				OS=Homo sapiens GN=ATAD3C PE=1 SV=2
169		RL18_HUMAN	55	21741				60S ribosomal protein L18 OS=Homo sapiens GN=RPL18 PE=1 SV=2
170		RLA1_HUMAN	54	11507				60S acidic ribosomal protein P1 OS=Homo sapiens GN=RPLP1 PE=1 SV=1
171		PGBM_HUMAN	54	468532				Basement membrane-specific heparan sulfate proteoglycan core protein OS=Homo sapiens GN=HSPG2 PE=1 SV=4
172		PTCD3_HUMAN	53	78651				Pentatricopeptide repeat-containing protein 3, mitochondrial OS=Homo sapiens GN=PTCD3 PE=1 SV=3
173		DDX21_HUMAN	52	87290				Nucleolar RNA helicase 2 OS=Homo sapiens GN=DDX21 PE=1 SV=5
174		GRP75_HUMAN	52	73778				Stress-70 protein, mitochondrial OS=Homo sapiens GN=HSPA9 PE=1 SV=2
175		RBM3_HUMAN	51	17160				Putative RNA-binding protein 3 OS=Homo sapiens GN=RBM3 PE=1 SV=1
176		IF4G1_HUMAN	51	175382				Eukaryotic translation initiation factor 4 gamma 1 OS=Homo sapiens GN=EIF4G1 PE=1 SV=4
177		H13_HUMAN	51	22352				Histone H1.3 OS=Homo sapiens GN=HIST1H1D PE=1 SV=2
178		RT09_HUMAN	50	45806				28S ribosomal protein S9, mitochondrial OS=Homo sapiens GN=MRPS9 PE=1 SV=2
179		RT35_HUMAN	50	36897				28S ribosomal protein S35, mitochondrial OS=Homo sapiens GN=MRPS35 PE=1 SV=1
180		RL24_HUMAN	48	17836				60S ribosomal protein L24 OS=Homo sapiens GN=RPL24 PE=1 SV=1
181		RL27_HUMAN	47	15840				60S ribosomal protein L27 OS=Homo sapiens GN=RPL27 PE=1 SV=2
182		RM12_HUMAN	47	21375				39S ribosomal protein L12, mitochondrial OS=Homo sapiens GN=MRPL12 PE=1 SV=2
183		ARF1_HUMAN	46	20684				ADP-ribosylation factor 1 OS=Homo sapiens GN=ARF1 PE=1 SV=2
184		H15_HUMAN	46	22578				Histone H1.5 OS=Homo sapiens GN=HIST1H1B PE=1 SV=3

185		SRS10_HUMAN	46	31282				Serine/arginine-rich splicing factor 10 OS=Homo sapiens GN=SRSF10 PE=1 SV=1
186		VIME_HUMAN	45	53791				Vimentin OS=Homo sapiens GN=VIM PE=1 SV=4
187		SF3B4_HUMAN	45	44425				Splicing factor 3B subunit 4 OS=Homo sapiens GN=SF3B4 PE=1 SV=1
188		ZFR_HUMAN	44	117162				Zinc finger RNA-binding protein OS=Homo sapiens GN=ZFR PE=1 SV=2
189		MYL6_HUMAN	44	16939				Myosin light polypeptide 6 OS=Homo sapiens GN=MYL6 PE=1 SV=2
190		RM44_HUMAN	43	37603				39S ribosomal protein L44, mitochondrial OS=Homo sapiens GN=MRPL44 PE=1 SV=1
191		U5S1_HUMAN	43	109530				116 kDa U5 small nuclear ribonucleoprotein component OS=Homo sapiens GN=EFTUD2 PE=1 SV=1
192		DEOC_HUMAN	41	35208				Putative deoxyribose-phosphate aldolase OS=Homo sapiens GN=DERA PE=1 SV=2
193		DESP_HUMAN	41	332410				Desmoplakin OS=Homo sapiens GN=DSP PE=1 SV=3
194		ATPA_HUMAN	41	59865				ATP synthase subunit alpha, mitochondrial OS=Homo sapiens GN=ATP5A1 PE=1 SV=1
195		ODBB_HUMAN	40	43179				2-oxoisovalerate dehydrogenase subunit beta, mitochondrial OS=Homo sapiens GN=BCKDHB PE=1 SV=2
196		RM49_HUMAN	40	19186				39S ribosomal protein L49, mitochondrial OS=Homo sapiens GN=MRPL49 PE=1 SV=1
197		PCBP1_HUMAN	40	37538				Poly(rC)-binding protein 1 OS=Homo sapiens GN=PCBP1 PE=1 SV=2
198		RT28_HUMAN	39	20830				28S ribosomal protein S28, mitochondrial OS=Homo sapiens GN=MRPS28 PE=1 SV=1
199		RS11_HUMAN	38	18475				40S ribosomal protein S11 OS=Homo sapiens GN=RPS11 PE=1 SV=3
200		RL5_HUMAN	37	34436				60S ribosomal protein L5 OS=Homo sapiens GN=RPL5 PE=1 SV=3
201		RUXF_HUMAN	37	9731				Small nuclear ribonucleoprotein F OS=Homo sapiens GN=SNRPF PE=1 SV=1
202		RS10_HUMAN	37	18958				40S ribosomal protein S10 OS=Homo sapiens GN=RPS10

								PE=1 SV=1
203		SF3B5_HUMAN	37	10145				Splicing factor 3B subunit 5 OS=Homo sapiens GN=SF3B5 PE=1 SV=1
204		DJC10_HUMAN	37	91021				DnaJ homolog subfamily C member 10 OS=Homo sapiens GN=DNAJC10 PE=1 SV=2
205		RENT1_HUMAN	37	124267				Regulator of nonsense transcripts 1 OS=Homo sapiens GN=UPF1 PE=1 SV=2
206		RBL1_HUMAN	36	121013				Retinoblastoma-like protein 1 OS=Homo sapiens GN=RBL1 PE=1 SV=3
207		PYR1_HUMAN	36	242829				CAD protein OS=Homo sapiens GN=CAD PE=1 SV=3
208		LSM12_HUMAN	36	21715				Protein LSM12 homolog OS=Homo sapiens GN=LSM12 PE=1 SV=2
209		ANXA2_HUMAN	36	38664				Annexin A2 OS=Homo sapiens GN=ANXA2 PE=1 SV=2
210		RL21_HUMAN	35	18609				60S ribosomal protein L21 OS=Homo sapiens GN=RPL21 PE=1 SV=2
211		RBMS1_HUMAN	35	44517				RNA-binding motif, single-stranded-interacting protein 1 OS=Homo sapiens GN=RBMS1 PE=1 SV=3
212		RAN_HUMAN	35	24444				GTP-binding nuclear protein Ran OS=Homo sapiens GN=RAN PE=1 SV=3
213		FSIP2_HUMAN	35	780119	1.323	1.037	2	Fibrous sheath-interacting protein 2 OS=Homo sapiens GN=FSIP2 PE=1 SV=4
214		RT07_HUMAN	35	28196				28S ribosomal protein S7, mitochondrial OS=Homo sapiens GN=MRPS7 PE=1 SV=2
215		RL11_HUMAN	35	20307				60S ribosomal protein L11 OS=Homo sapiens GN=RPL11 PE=1 SV=2
216		RL26_HUMAN	34	17248				60S ribosomal protein L26 OS=Homo sapiens GN=RPL26 PE=1 SV=1
217		SPTN1_HUMAN	34	284958				Spectrin alpha chain, non-erythrocytic 1 OS=Homo sapiens GN=SPTAN1 PE=1 SV=3
218		SC31A_HUMAN	34	133091				Protein transport protein Sec31A OS=Homo sapiens GN=SEC31A PE=1 SV=3
219		RSSA_HUMAN	33	32833				40S ribosomal protein SA OS=Homo sapiens GN=RPSA PE=1 SV=4
220		G3P_HUMAN	32	36030				Glyceraldehyde-3-phosphate dehydrogenase OS=Homo sapiens GN=GAPDH PE=1 SV=3

221		RS26L_HUMAN	32	12994				Putative 40S ribosomal protein S26-like 1 OS=Homo sapiens GN=RPS26P11 PE=5 SV=1
222		IF4A1_HUMAN	31	46125				Eukaryotic initiation factor 4A-I OS=Homo sapiens GN=EIF4A1 PE=1 SV=1
223		EBP2_HUMAN	31	34830				Probable rRNA-processing protein EBP2 OS=Homo sapiens GN=EBNA1BP2 PE=1 SV=2
224		RT23_HUMAN	30	21825				28S ribosomal protein S23, mitochondrial OS=Homo sapiens GN=MRPS23 PE=1 SV=2
225		DSG1_HUMAN	30	113676				Desmoglein-1 OS=Homo sapiens GN=DSG1 PE=1 SV=2
226		RL38_HUMAN	30	8233				60S ribosomal protein L38 OS=Homo sapiens GN=RPL38 PE=1 SV=2
227		KV116_HUMAN	29	11787				Ig kappa chain V-I region Roy OS=Homo sapiens PE=1 SV=1
228		ERH_HUMAN	29	12251				Enhancer of rudimentary homolog OS=Homo sapiens GN=ERH PE=1 SV=1
229		PCBP2_HUMAN	29	38611				Poly(rC)-binding protein 2 OS=Homo sapiens GN=PCBP2 PE=1 SV=1
230		RT02_HUMAN	29	33332				28S ribosomal protein S2, mitochondrial OS=Homo sapiens GN=MRPS2 PE=1 SV=1
231		PECR_HUMAN	29	32560				Peroxisomal trans-2-enoyl-CoA reductase OS=Homo sapiens GN=PECR PE=1 SV=2
232		SF3A1_HUMAN	29	88970				Splicing factor 3A subunit 1 OS=Homo sapiens GN=SF3A1 PE=1 SV=1
233		RPN2_HUMAN	28	69241				Dolichyl-diphosphooligosaccharide--protein glycosyltransferase subunit 2 OS=Homo sapiens GN=RPN2 PE=1 SV=3
234		OTOF_HUMAN	28	227096				Otoferlin OS=Homo sapiens GN=OTOF PE=1 SV=3
235		SF01_HUMAN	28	68286				Splicing factor 1 OS=Homo sapiens GN=SF1 PE=1 SV=4
236		PMFBP_HUMAN	28	119129				Polyamine-modulated factor 1-binding protein 1 OS=Homo sapiens GN=PMFBP1 PE=2 SV=1
237		CELR1_HUMAN	27	329278	1044	20.23	2	Cadherin EGF LAG seven-pass G-type receptor 1 OS=Homo sapiens GN=CELSR1 PE=1 SV=1
238		DYH14_HUMAN	27	400222				Dynein heavy chain 14, axonemal OS=Homo sapiens GN=DNAH14 PE=2 SV=3
								60S ribosomal protein L13a

239		RL13A_HUMAN	27	23650				OS=Homo sapiens GN=RPL13A PE=1 SV=2
240		ZN326_HUMAN	26	65613				DBIRD complex subunit ZNF326 OS=Homo sapiens GN=ZNF326 PE=1 SV=2
241		TRI11_HUMAN	26	52741				E3 ubiquitin-protein ligase TRIM11 OS=Homo sapiens GN=TRIM11 PE=1 SV=2
242		RL37A_HUMAN	26	10296				60S ribosomal protein L37a OS=Homo sapiens GN=RPL37A PE=1 SV=2
243		RPKL1_HUMAN	26	59999	**1.466**	**1.001**	**2**	Ribosomal protein S6 kinase-like 1 OS=Homo sapiens GN=RPS6KL1 PE=2 SV=1
244		RL35_HUMAN	26	14602				60S ribosomal protein L35 OS=Homo sapiens GN=RPL35 PE=1 SV=2
245		KDM8_HUMAN	25	47240	5.633e+4	4.532	2	Lysine-specific demethylase 8 OS=Homo sapiens GN=KDM8 PE=1 SV=1
246		TYW3_HUMAN	25	29775	**2.443e+4**	**1.470**	**2**	tRNA wybutosine-synthesizing protein 3 homolog OS=Homo sapiens GN=TYW3 PE=2 SV=2
247		WIPI2_HUMAN	25	49377				WD repeat domain phosphoinositide-interacting protein 2 OS=Homo sapiens GN=WIPI2 PE=1 SV=1
248		PDE3A_HUMAN	25	125160				cGMP-inhibited 3',5'-cyclic phosphodiesterase A OS=Homo sapiens GN=PDE3A PE=1 SV=3
249		NOP2_HUMAN	25	89455				Putative ribosomal RNA methyltransferase NOP2 OS=Homo sapiens GN=NOP2 PE=1 SV=2
250		RL35A_HUMAN	24	12582				60S ribosomal protein L35a OS=Homo sapiens GN=RPL35A PE=1 SV=2
251		CL029_HUMAN	24	37506				Uncharacterized protein C12orf29 OS=Homo sapiens GN=C12orf29 PE=1 SV=2
252		NNTM_HUMAN	24	113918				NAD(P) transhydrogenase, mitochondrial OS=Homo sapiens GN=NNT PE=1 SV=3
253		CPSF2_HUMAN	24	88431				Cleavage and polyadenylation specificity factor subunit 2 OS=Homo sapiens GN=CPSF2 PE=1 SV=2
254		KV401_HUMAN	23	13384				Ig kappa chain V-IV region (Fragment) OS=Homo sapiens GN=IGKV4-1 PE=4 SV=1
255		PRP19_HUMAN	22	55146				Pre-mRNA-processing factor 19 OS=Homo sapiens GN=PRPF19 PE=1 SV=1
256		TBK1_HUMAN	22	83589				Serine/threonine-protein kinase TBK1 OS=Homo sapiens GN=TBK1 PE=1 SV=1

257		CND3_HUMAN	22	114461				Condensin complex subunit 3 OS=Homo sapiens GN=NCAPG PE=1 SV=1
258		PLCL2_HUMAN	22	125981				Inactive phospholipase C-like protein 2 OS=Homo sapiens GN=PLCL2 PE=1 SV=2
259		RT24_HUMAN	21	19055				28S ribosomal protein S24, mitochondrial OS=Homo sapiens GN=MRPS24 PE=1 SV=1
260		2ABA_HUMAN	21	51771				Serine/threonine-protein phosphatase 2A 55 kDa regulatory subunit B alpha isoform OS=Homo sapiens GN=PPP2R2A PE=1 SV=1
261		RT31_HUMAN	21	45402				28S ribosomal protein S31, mitochondrial OS=Homo sapiens GN=MRPS31 PE=1 SV=3
262		RS30_HUMAN	21	6672				40S ribosomal protein S30 OS=Homo sapiens GN=FAU PE=1 SV=1
263		TDRD7_HUMAN	21	123658				Tudor domain-containing protein 7 OS=Homo sapiens GN=TDRD7 PE=1 SV=2
264		DYH9_HUMAN	21	511551				Dynein heavy chain 9, axonemal OS=Homo sapiens GN=DNAH9 PE=1 SV=3
265		FIGL1_HUMAN	21	74154				Fidgetin-like protein 1 OS=Homo sapiens GN=FIGNL1 PE=1 SV=2
266		BRX1_HUMAN	21	41487				Ribosome biogenesis protein BRX1 homolog OS=Homo sapiens GN=BRIX1 PE=1 SV=2
267		MOV10_HUMAN	21	113895				Putative helicase MOV-10 OS=Homo sapiens GN=MOV10 PE=1 SV=2
268		TCPH_HUMAN	20	59437				T-complex protein 1 subunit eta OS=Homo sapiens GN=CCT7 PE=1 SV=2
269		IDE_HUMAN	20	118077				Insulin-degrading enzyme OS=Homo sapiens GN=IDE PE=1 SV=4
270		SPTN5_HUMAN	20	416579				Spectrin beta chain, non-erythrocytic 5 OS=Homo sapiens GN=SPTBN5 PE=1 SV=1
271		CENPP_HUMAN	20	33224				Centromere protein P OS=Homo sapiens GN=CENPP PE=1 SV=1
272		GAR1_HUMAN	19	22434				H/ACA ribonucleoprotein complex subunit 1 OS=Homo sapiens GN=GAR1 PE=1 SV=1
273		RL36_HUMAN	18	12310				60S ribosomal protein L36 OS=Homo sapiens GN=RPL36 PE=1 SV=3
								Signal-induced proliferation-associated 1-like protein 1

274		SI1L1_HUMAN	18	200371				OS=Homo sapiens GN=SIPA1L1 PE=1 SV=4
275		SYNE2_HUMAN	18	797092				Nesprin-2 OS=Homo sapiens GN=SYNE2 PE=1 SV=3
276		PSA2_HUMAN	18	25926				Proteasome subunit alpha type-2 OS=Homo sapiens GN=PSMA2 PE=1 SV=2
277		STAT3_HUMAN	18	88011	**272.7**	**1.096**	**2**	Signal transducer and activator of transcription 3 OS=Homo sapiens GN=STAT3 PE=1 SV=2
278		NP1L2_HUMAN	18	52577	**0.01490**	**1.915**	**3**	Nucleosome assembly protein 1-like 2 OS=Homo sapiens GN=NAP1L2 PE=2 SV=1
279		ZDH17_HUMAN	18	72688				Palmitoyltransferase ZDHHC17 OS=Homo sapiens GN=ZDHHC17 PE=1 SV=2
280		RL28_HUMAN	17	15817				60S ribosomal protein L28 OS=Homo sapiens GN=RPL28 PE=1 SV=3
281		RL10_HUMAN	17	24588				60S ribosomal protein L10 OS=Homo sapiens GN=RPL10 PE=1 SV=4
282		RPOM_HUMAN	17	138907				DNA-directed RNA polymerase, mitochondrial OS=Homo sapiens GN=POLRMT PE=1 SV=2
283		CP071_HUMAN	17	55779				Uncharacterized protein C16orf71 OS=Homo sapiens GN=C16orf71 PE=2 SV=3
284		RM04_HUMAN	17	34897				39S ribosomal protein L4, mitochondrial OS=Homo sapiens GN=MRPL4 PE=1 SV=1
285		LRMP_HUMAN	17	62083				Lymphoid-restricted membrane protein OS=Homo sapiens GN=LRMP PE=1 SV=3
286		PRC2B_HUMAN	16	243471				Protein PRRC2B OS=Homo sapiens GN=PRRC2B PE=1 SV=2
287		RET7_HUMAN	16	15554				Retinoid-binding protein 7 OS=Homo sapiens GN=RBP7 PE=1 SV=1
288		POF1B_HUMAN	16	68134				Protein POF1B OS=Homo sapiens GN=POF1B PE=1 SV=3
289		CRML_HUMAN	15	134931				Protein cramped-like OS=Homo sapiens GN=CRAMP1L PE=1 SV=3
290		ST32C_HUMAN	15	54960				Serine/threonine-protein kinase 32C OS=Homo sapiens GN=STK32C PE=1 SV=1
291		CARM1_HUMAN	15	65899				Histone-arginine methyltransferase CARM1 OS=Homo sapiens GN=CARM1 PE=1 SV=3
								Low-density lipoprotein

292		LRP2_HUMAN	15	521616				receptor-related protein 2 OS=Homo sapiens GN=LRP2 PE=1 SV=3
293		VSIG4_HUMAN	15	44015				V-set and immunoglobulin domain-containing protein 4 OS=Homo sapiens GN=VSIG4 PE=1 SV=1
294		PCX1_HUMAN	14	259040				Pecanex-like protein 1 OS=Homo sapiens GN=PCNX PE=2 SV=2
295		BDP1_HUMAN	14	294339				Transcription factor TFIIIB component B'' homolog OS=Homo sapiens GN=BDP1 PE=1 SV=3
296		NALP1_HUMAN	13	165760				NACHT, LRR and PYD domains-containing protein 1 OS=Homo sapiens GN=NLRP1 PE=1 SV=1
297		PRIP1_HUMAN	13	71970				ProSAP-interacting protein 1 OS=Homo sapiens GN=PROSAPIP1 PE=2 SV=1
298		M3K9_HUMAN	13	122126				Mitogen-activated protein kinase kinase kinase 9 OS=Homo sapiens GN=MAP3K9 PE=1 SV=3
