# Supplementary material for: Inhibition of nucleolar stress response by Sirt1: A potential mechanism of acetylation‐independent regulation of p53 accumulation
Source: Aging Cell. 2019 Jan 8;18(2):e12900. doi: 10.1111/acel.12900 (PMC6413664; doi:10.1111/acel.12900)
Supplement: Supplementary file 4 [file ACEL-18-e12900-s004.pdf]

Supplemental Methods

Plasmids

Plasmids expressing wild type human Sirt1 (pECE-FLAG-Sirt1) and the enzyme-dead Sirt1 mutant (pECE-FLAG-Sirt1/H363Y) as described were obtained from Addgene (Brunet *et al.*, 2004). pGEX-4T-1 vector was purchased from GE Healthcare (Pittsburgh, PA, USA). peGFP-p53 was a gift from Geoff Wahl (Addgene plasmid # 11770). peGFP/FLAG-NPM (expressing N-terminal eGFP/FLAG-tagged wild type NPM fusion protein) was from Addgene (Wang *et al.*, 2005). The cDNA of p53 mutant (p53/8KR) (Tang *et al.*, 2008), in which all of the eight acetylated lysine residues were substituted with arginine, was constructed by chemical gene synthesis (Biosune, Shanghai, China) and inserted into the peGFP-C3 vector. Sirt1 deletion mutants were constructed by either overlap primer extension PCR or inverse PCR as described (Ho *et al.*, 1989; Imai *et al.*, 1991), using pECE-FLAG-Sirt1 as the template. NPM mutants with K223 changed to Q or R (pNPM-K223Q/R) were constructed using QuikChange Site-Directed Mutagenesis Kit from Agilent Technologies (Santa Clara, CA, USA).

Antibodies

The following antibodies were purchased from Abcam (Cambridge, UK): NPM (ab10530), nucleostemin (ab70346), histone H3 (ab1791), fibrillarin (ab5821), GFP (ab13970). The following antibodies were purchased from Cell Signaling (Beverley, MA, USA): p53 (#2524), Sirt1 (#2496), DDDDK tag (FLAG) (#2368). Anti-acetylated lysine (clone 15G10) was from BioLegend (#623402) (San Diego, CA, USA). Anti-DDDDK conjugated agarose beads were purchased from MBL (PM020-8) (Woburn, MA, USA) and OriGene (#TA150037) (Rockville, MD, USA).

Cell culture and transfections

HeLa cells were maintained in high-glucose DMEM (Thermo Fisher Scientific, Waltham, MA, USA). Human umbilical vein endothelial cells (HUVECs) were cultured in complete ECM medium (ScienCell, Carlsbad, CA, USA) as described (Liu *et al.*, 2015). Human aortic smooth muscle cells (HASMCs) (ATCC #PCS-100-012) were cultured in complete SMCM medium as indicated by the manufacturer (ScienCell, Carlsbad, CA, USA). Cells were maintained in the presence of penicillin (10 U/ml) and streptomycin (10 μg/ml) in 5% CO_2. Plasmid and siRNA transfections were performed using Lipofectamine LTX or Lipofectamine RNAiMAX respectively (all from Thermo Fisher Scientific, Waltham, MA, USA) as described previously (Liu *et al.*, 2015).

SILAC and mass spectrometry analysis

Stable isotope labeling by amino acids in cell culture (SILAC) technique was used to identify Sirt1-binding partners (Gruhler & Kratchmarova, 2008). Labeled HeLa cells were continuously passaged for 6 times in SILAC DMEM medium supplemented with $^{13}C/^{15}N$-labeled (heavy) arginine (Thermo). Non-labeled cells were transfected with Flag-Sirt1 and maintained in normal DMEM. Labeled and non-labeled cells of equal number were mixed and homogenized using an ultrasonic disruptor. Total protein was extracted and immunoprecipitated with anti-DDDDK agarose beads. Digested peptides were separated by nanoscale liquid chromatography, followed by mass spectrometry analysis using an ESI-LTQ-Orbitrap Velos Pro system (Thermo Fisher). The proteomic data was processed using MASCOT Distiller (Matrix Science, Boston, MA, USA).

Purification of nucleoli

Nucleoli purification was performed according to the protocol descried by Lam and Lamond (Lam & Lamond, 2006). Briefly, 10^7 HeLa cells were harvested by trypsin digestion and

washed with cold PBS. The cells were resuspended in 5 ml of Buffer A (10 mM Hepes, pH 7.9, 10 mM KCl, 1.5 mM $MgCl_2$, 0.5 mM DTT) for a hypotonic shock treatment for 30 min. Then the cells were disrupted using a Dounce homogenizer till more than 90% of the cells were broken by checking under a phase contrast microscope. The nuclei were collected by centrifugation (200×g for 5 min) and resuspended in 3 ml of S1 solution (0.25 M sucrose, 10 mM $MgCl_2$). The crude nuclei fraction was then layered on 3 ml of S2 solution (0.35 M sucrose, 0.5 mM $MgCl_2$), centrifuged at 1400×g for 5 min. The pelleted nuclei were resuspended in 3 ml of S2 solution and sonicated for 6 intermittent 10-second bursts at 60% power. The homogenate was layered on 3 ml of S3 solution (0.88 M sucrose, 0.5 mM $MgCl_2$) and centrifuged at 2000×g for 10min to pellet the nucleoli, which were washed and finally collected in 0.5 ml of S2 solution. All procedures were carried out on ice.

Immunofluorescence and confocal microscopy

Immunofluorescence was performed as described previously (Liu *et al.*, 2015). Cells cultured on Lab-Tek II chamber slides were fixed in 4% paraformaldehyde, permeabilized with 0.5% Triton X-100, and blocked with 2% BSA for 30 min. Cells were then incubated with primary antibodies overnight followed by conjugated anti-IgG (Jackson ImmunoResearch Laboratories, West Grove, PA, USA) for 1 h. DAPI was used for counterstaining. Images were taken with a laser-scanning confocal microscope (Model LSM710, Zeiss, Jena, Germany). To assess NSR, fluorescent images for NPM were reviewed by an independent investigator and the nuclei were classified as either nucleoli-intact or nucleoli-disrupted in a blind manner. We also measured the average florescence intensity of nucleoli and nucleoplasm using Image J software (NIH), and used the nucleoplasm to nucleoli florescence intensity ratio as an index of nucleolar disruption. For each experiment, 10 random high-power fields or at least 50 cells from different

areas were examined.

Immunoprecipitation and western blot

Cells were homogenized by sonication in cold lysis buffer. The lysates were precleared using non-immune IgG, and incubated with 2 μg of capture antibody and 20 μl of 50% protein A/G-agarose bead slurry (Pierce Biotechnology, Rockford, IL, USA) overnight at 4 °C with gentle rotation. The beads were washed and boiled in 2 × Laemmli buffer. Western blot was performed as described (Yan *et al.*, 2014).

Real-time quantitative PCR

Total RNA was extracted using TRIzol (Thermo) and quantified with NanoDrop 2000 (Thermo). Reverse transcription and quantitative PCR (qPCR) were performed using PrimeScript and SYBR Premix Ex Taq kits respectively (all from Takara, Dalian, China). The primer sequences for human pre-rRNA were GCCTTCTCTAGCGATCTGAGAG (forward) and CCATAACGGAGGCAGAGACA (reverse) as described (Liao *et al.*, 2014). The mRNA of β-actin was used as the house-keeping gene. The $2^{-\Delta\Delta Ct}$ method was used to assess the relative RNA expression levels.

Preparation of recombinant NPM

Human NPM cDNA as PCR amplified and cloned into pGEX-4T-1 at the BamHI site, and used to transformed BL21 competent cells. Single clones were recovered and the correct insertion was verified by sequencing. Cells were cultured and expression induced by IPTG for 1 hr. The fusion protein was purified using glutathione spin columns (from Thermo) and verified by SDS-PAGE and Coomassie Brilliant Blue staining.

In vitro deacetylation assay

Purified GST-NPM bound on the resin bead was mixed with 1 μg of recombinant HAT

domain of the human CBP acetyltransferase (from Abcam), in 8 ml of acetylation buffer containing 100 μM acetyl-CoA, 50 mM Tris (pH 8.0), 100 mM NaCl, 10% glycerol, 0.1 mM EDTA, 1 mM DTT, 1 mM PMSF and 5 mM sodium butyrate as described (Daitoku *et al.*, 2004). The beads were incubated at 30°C for 3 hr with rotation, and washed with PBS. Half of the beads were recovered and then incubated with 5 μg of recombinant human Sirt1 protein in a deacetylation reaction buffer containing 1 mM oxidized nicotinamide adenine dinucleotide (NAD^+), 50 mM Tris (pH9.0), 50 mM NaCl, 4 mM $MgCl_2$, 0.5 mM DTT, 0.2 mM PMSF, 0.02% NP-40 and 5% glycerol, at 30°C for 3 hr. The bound fusion protein was eluted using reduced glutathione. The acetylation status of different lysine residues was characterized by mass spectrometry.

Live cell imaging

HeLa cells expressing eGFP-NPM were continuously monitored with a spinning-disk confocal live cell imaging system (Cell Observer SD, Zeiss) equipped with a top cage incubator. Fluorescent photographs were taken every 15 min for a total period of 24 hr, with a laser excitation at 490 nm.

Flow cytometry analysis

Cell cycle was analyzed using a FACSCalibur cytometer (BD Biosciences, Mountain View, CA, USA). Cells were detached with trypsin and fixed overnight in cold ethanol. Propidium iodide staining was performed using a kit from Abcam (ab139418) according to the manufacturer's instructions.

Statistics

Data are presented as mean ± standard error of the mean (SEM). Data analysis was performed using unpaired *t*-test or one-way analysis of variance (ANOVA) followed by *post hoc*

Tukey's test as appropriate. *P* < 0.05 was considered as statistically significant.

References

Brunet, A., Sweeney, L. B., Sturgill, J. F., Chua, K. F., Greer, P. L., Lin, Y., Tran, H., et al. (2004). Stress-dependent regulation of FOXO transcription factors by the SIRT1 deacetylase. *Science, 303*(5666), 2011-2015. https://doi: 10.1126/science.1094637. 1094637 [pii]

Daitoku, H., Hatta, M., Matsuzaki, H., Aratani, S., Ohshima, T., Miyagishi, M., Nakajima, T., et al. (2004). Silent information regulator 2 potentiates Foxo1-mediated transcription through its deacetylase activity. *Proc Natl Acad Sci U S A, 101*(27), 10042-10047. https://doi: 10.1073/pnas.0400593101. 0400593101 [pii]

Gruhler, S., & Kratchmarova, I. (2008). Stable isotope labeling by amino acids in cell culture (SILAC). *Methods Mol Biol, 424*(101-111. https://doi: 10.1007/978-1-60327-064-9_9

Ho, S. N., Hunt, H. D., Horton, R. M., Pullen, J. K., & Pease, L. R. (1989). Site-directed mutagenesis by overlap extension using the polymerase chain reaction. *Gene, 77*(1), 51-59. https://doi: 0378-1119(89)90358-2 [pii]

Imai, Y., Matsushima, Y., Sugimura, T., & Terada, M. (1991). A simple and rapid method for generating a deletion by PCR. *Nucleic Acids Res, 19*(10), 2785. https://doi:

Lam, Y. W., & Lamond, A. I. (2006). Isolation of Nucleoli. In J. E. Celis (Ed.), *Cell Biology - A Laboratory Handbook (Third Edition)* (3 ed., Vol. 2, pp. 103-107): Elsevier.

Liao, P., Wang, W., Shen, M., Pan, W., Zhang, K., Wang, R., Chen, T., et al. (2014). A positive feedback loop between EBP2 and c-Myc regulates rDNA transcription, cell proliferation, and tumorigenesis. *Cell Death Dis, 5*(e1032. https://doi: cddis2013536 [pii]10.1038/cddis.2013.536

Liu, J., Bi, X., Chen, T., Zhang, Q., Wang, S. X., Chiu, J. J., Liu, G. S., et al. (2015). Shear stress regulates endothelial cell autophagy via redox regulation and Sirt1 expression. *Cell Death Dis, 6*(e1827. https://doi: cddis2015193 [pii]10.1038/cddis.2015.193

Tang, Y., Zhao, W., Chen, Y., Zhao, Y., & Gu, W. (2008). Acetylation is indispensable for p53 activation. *Cell, 133*(4), 612-626. https://doi: S0092-8674(08)00441-8 [pii]10.1016/j.cell.2008.03.025

Wang, W., Budhu, A., Forgues, M., & Wang, X. W. (2005). Temporal and spatial control of nucleophosmin by the Ran-Crm1 complex in centrosome duplication. *Nat Cell Biol, 7*(8), 823-830. https://doi: ncb1282 [pii]10.1038/ncb1282

Yan, F., Wang, Y., Wu, X., Peshavariya, H. M., Dusting, G. J., Zhang, M., & Jiang, F. (2014). Nox4 and redox signaling mediate TGF-beta-induced endothelial cell apoptosis and phenotypic switch. *Cell Death Dis, 5*(e1010. https://doi: cddis2013551 [pii]10.1038/cddis.2013.551
